# Microbial Community Structure of Deep-sea Hydrothermal Vents on the Ultraslow Spreading Southwest Indian Ridge

**DOI:** 10.3389/fmicb.2017.01012

**Published:** 2017-06-13

**Authors:** Jian Ding, Yu Zhang, Han Wang, Huahua Jian, Hao Leng, Xiang Xiao

**Affiliations:** ^1^School of Life Science and Biotechnology, Shanghai Jiao Tong UniversityShanghai, China; ^2^Institute of Oceanography, Shanghai Jiao Tong UniversityShanghai, China

**Keywords:** ultraslow-spreading ridge, SWIR, hydrothermal vent, 16S ribosomal RNA, microbial community

## Abstract

Southwest Indian Ridge (SWIR) is a typical oceanic ultraslow spreading ridge with intensive hydrothermal activities. The microbial communities in hydrothermal fields including primary producers to support the entire ecosystem by utilizing geochemical energy generated from rock-seawater interactions. Here we have examined the microbial community structures on four hydrothermal vents from SWIR, representing distinct characteristics in terms of temperature, pH and metal compositions, by using Illumina sequencing of the 16S small subunit ribosomal RNA (rRNA) genes, to correlate bacterial and archaeal populations with the nature of the vents influenced by ultraslow spreading features. *Epsilon-, Gamma-, Alpha*-, and *Deltaproteobacteria* and members of the phylum *Bacteroidetes* and *Planctomycetes*, as well as *Thaumarchaeota, Woesearchaeota*, and *Euryarchaeota* were dominant in all the samples. Both bacterial and archaeal community structures showed distinguished patterns compared to those in the fast-spreading East Pacific Ridge or the slow-spreading Mid-Atlantic Ridge as previously reported. Furthermore, within SWIR, the microbial communities are highly correlated with the local temperatures. For example, the sulfur-oxidizing bacteria were dominant within bacteria from low-temperature vents, but were not represented as the dominating group recovered from high temperature (over 300°C) venting chimneys in SWIR. Meanwhile, *Thaumarchaeota*, the ammonium oxidizing archaea, only showed high relative abundance of amplicons in the vents with high-temperature in SWIR. These findings provide insights on the microbial community in ultraslow spreading hydrothermal fields, and therefore assist us in the understanding of geochemical cycling therein.

## Introduction

Hydrothermal venting is one of the fundamental processes by which heat and chemical species are transferred from the lithosphere to the ocean, and venting occurs along divergent plate boundaries in every ocean, at all spreading rates, and in a diversity of geological settings (Baker and German, 2004). More than 300 seafloor vent fields have been investigated in diverse settings spanning oceanic ridges, volcanic arcs, and hot spots (Corliss et al., 1979; Hannington et al., 2011). Considering that hydrothermal fluids emanate from the subsurface, these environments are considered “windows into the subseafloor” (Reveillaud et al., 2016). According to the disquisitive descriptions on the spreading rates, the ocean ridges have been divided into fast- (~80–180 mm year^−1^ full rate), intermediate- (~55–70 mm year^−1^), slow-(less than 55 mm year^−1^), and ultraslow- (less than 20 mm year^−1^) spreading ridges (Dick et al., 2003; Ehlers and Jokat, 2009). Most interest in the mid-oceanic ridges has been focused on hydrothermal activities in the fast-, slow-, and intermediate-spreading ridges (Hannington et al., 2005). In January–March 2007, the Chinese research cruise DY115-19 discovered an active hydrothermal field, the 49°39′E field (6 mm year^−1^) on SWIR during the Chinese research cruise DY115-19 (Zhu et al., 2010; Tao et al., 2012). Before this discovery, only the Gakkel Ridge was discovered on global ultra-slow spreading ridges (Connelly et al., 2007). The Gakkel Ridge ranged from 7°W to 86°E (6–11 mm year^−1^) and held numerous anomalies of the hydrothermal activity (Edmonds et al., 2003). The SWIR separates the African and Antarctic plates, extends from the east Rodriguez triple junction (RTJ) to the west Bouvet triple junction (BTJ), and spreads at a full rate of 14 mm year^−1^ (Sauter and Cannat, 2010). The Longqi vent field at 49°39′E, 37°47′S on SWIR was discovered and recognized as the first active field found on this ultraslow spreading ridge (Zhu et al., 2010). The surrounding area is basaltic-hosted environments and lacking sediments. According to the previous study of proposed modes for Longqi field, the significantly thinned crust was observed. This suggested that the tectonics were probably characterized by the early stage of the detachment fault in the area, which provided pathways for hydrothermal circulation period. Within Longqi hydrothermal field, three venting areas, the Vent S, M, and N, have been confirmed (Tao et al., 2012). This site offered new and exciting prospects for expanding the known ranges of minerals, fluids, biodiversity, and hydrothermal deposits at ultraslow-spreading ridge (Peng et al., 2011).

Deep-sea hydrothermal vents are some of the most biologically productive ecosystems on the Earth, yet receive little to no input of organic matter derived photosynthetically (Rutherford, 2014). The ecosystems at hydrothermal vents host complex, dynamic habitats characterized by steep gradients in temperature and geochemistry (Jannasch and Mottl, 1985). In the ridge habitats, the permeable mineral structures, and the continued mixing of chemically-reduced, vent-derived fluids with oxidized seawater provides favorable conditions that support the growth of microbial communities (Frank et al., 2013). Chemoautotrophs inhabiting these areas act as important primary producers, transferring the energy from the geothermal source to the higher trophic levels through several important microbial chemosynthetic pathways such as sulfur-oxidation, nitrification, etc. (Sievert and Vetriani, 2012). However, most of our knowledge on the microbial communities in hydrothermal vents has come from fast-spreading ridges, such as East Pacific Rise (EPR) (Gaill et al., 1987; Sylvan et al., 2012) and the slow-spreading Mid-Atlantic Ridge (MAR) (Flores et al., 2011). The distributional patterns of the microorganisms that colonize deep-sea hydrothermal vent chimneys at ultraslow-spreading ridge and their link to the geologic setting remain poorly understood, partly because of sampling limits. In this study, we had a chance to obtain environmental samples on/off chimney in SWIR by *Jiao Long* manned submersible and applied high-through sequencing on 16S rRNA genes. In this case, we could perform detailed analysis on the microbial communities and link them with their local habitat.

## Materials and methods

### Sample collection and description

**Figure 1:**
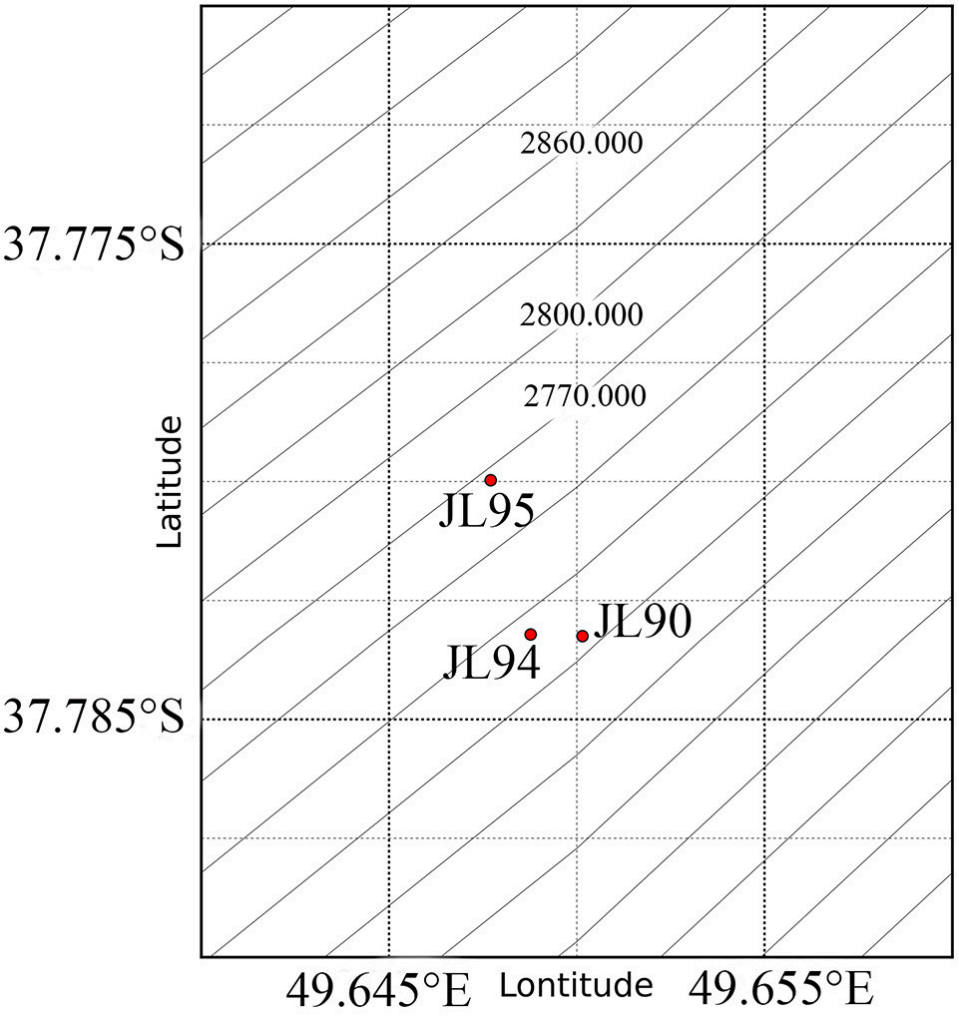
Schematic location of the sampling sites and Longqi vent Field (red Pentagram) at SWIR. The map was created using python.

Low- and high-temperature chimney samples were collected during November 2014-January 2015 Dayang 35 cruise to the Longqi field by using the manned submersible *Jiao Long* (Figure 1, Table 1). Longqi is a large, deep-sea hydrothermal venting field with approximately 6.7 × 10^4^ m^2^ of low-magnetization zone on the ultraslow spreading SWIR. After sampling by the robotic arm, the sample was sealed in bio-box which was prefilled with sterilized sea water to minimize contamination. The temperature of selected chimneys' venting fluid in this study was measured in a range of 13.3°C to 379°C. Immediately after the sample was brought onboard, it was stored at −20°C and later at −80°C in laboratory until further analysis. Comparisons were made to bacterial and archaeal sequence data of chimney samples collected from M vent on EPR9-10°N during the AT26-10 cruise from Dec 29, 2013 to Jan 26, 2014), and published data of LS7 vent on MAR obtained from NCBI SRA database (SRP005280).

**Table 1:** Concentrations of Elements in chimney deposits and physicochemical characteristics of their hydrothermal fluids at the Longqi field on SWIR.

**Chimneys**	**JL90**	**JL94D**	**JL94H**	**JL95**	**Range**
**ELEMENT(mg kg^−1^)**					
Fe	261,600	305,900	286,500	68,780	
S	300,200	394,400	302,400	1,682	
Zn	211,200	233,100	2,938	154	
Mn	3,786	33	14	343,800	
Mg	20,960	24	324	13,720	
Ca	9,268	112	46,000	13,140	
Pb	1,417	703	57	41	
Cu	806.8	541	67,330	42	
Al	661.6	5,680	141	429	
Depth (m)	2,746	2,768	2,778	2,775	
Location	49.6501525°E, 37.7832506°S	49.6487677°E, 37.7832151°S	49.6487677°E, 37.7832151°S	49.6477092°E, 37.7799720°S	
**FLUID**					
Max. Temp. (°C)	145	13.3	362	379	13.3–379
pH	4.85	–	–	3.42	3.21–4.85
Salinity	4.0	–	–	4.0	3.7–4.5
DO (mg L^−1^)	24.7	–	–	14.3	0.5–24.7

### DNA extraction, amplification, sequencing, and evaluation

For chimney samples, named as JL90, JL94D, JL94H, and JL95 from SWIR, and CH7 from EPR9°N (Figure S1). DNA extraction was only applied on their outer layers, approximately 1–5 mm of thickness, following the protocols described in detail previously (Zhou et al., 1996; Vengadesh et al., 2016). The 16S rRNA genes were amplified using the polymerase chain reaction (PCR) with primer sets 515F (5′-xxxxxxxGTGCCAGCMGCCGCGGTAA-3′, x region represents the tag for Miseq sequencing) and 806R (5′-GGACTACHVGGGTWTCTAAT-3′) for bacteria (De Mandal et al., 2015) and 519F (5′-xxxxxxxCAGYMGCCRCGGKAAHACC-3′) (Flores et al., 2011) and 915R (5′-RGTGCYCCCCCGCCAATTC-3′) (Pinto and Raskin, 2012) for archaea. The PCR condition for bacterial 16S rRNA genes amplification was: 95°C for 5 min; 30 cycles of 95°C for 40 s, 55°C for 40 s, 72°C for 40 s; 72°C for 10 min, and hold at 4°C. The PCR condition for archaeal 16S rRNA genes amplification was 95°C for 5 min; 35 cycles of 95°C for 40 s, 57°C for 40 s, 72°C for 1 min; 72°C for 10 min, and hold at 4°C. PCR products were purified using the Gel purification kit (Tiangen Biotech Co. Ltd., Beijing) before sending for Miseq sequencing in Personalbio Company (Shanghai). The assembled Miseq sequences were submitted into NCBI database (GenBank Accession No. PRJNA320664).

### Data analysis on the microbial communities

Data analysis of the 16S rRNA Miseq sequence was performed using QIIME version 1.9.1 software pipeline (Caporaso et al., 2010) and the QIIME-compatible version of the SILVA-123 database (https://www.arb-silva.de/) for template-based alignment and taxonomic assignment. Assembled reads that passed the chimera checking were clustered into *de novo* operational taxonomic units (OTUs) at a cut off of 97% sequence similarity. We calculated diversity indices (Shannon and chao1 evenness) using QIIME, and constructed distance matrices in PAST software package (Hammer et al., 2009) using the Bray-Curtis calculator of community membership and structure for comparison between samples. The Venn diagram and RDA analysis were carried by R Venn Diagram and Vegan package, respectively.

### Mineralogical analysis of hydrothermal chimneys

Mineral analysis of the chimney samples was obtained using X-Ray Fluorescence Spectrometer (XRF-1800, Shimadzu Japan), X-Ray Polycrystaline Diffractometer (D8 Advance, Bruker, German) and ICP-AES (iCAP6300, Thermo, USA). Powder XRF and XRD samples were dried overnight at 70°C and deposited on sample holders. The procedure for XRF and XRD analysis was carried out according to the methods described in details by Peng et al. (2011). Subsamples of materials used for the above analysis were collected for ICP-AES analysis; about 200 mg of this powdered and dried material from each sample was added to in a Teflon digestion-crucible and followed by a strong acid digestion method (Wong and Li, 2004).

## Results

### Habitat chemistry

The temperature of fluids at sampling vent sites ranged from a low temperature of 13.3°C for JL90 to a high-temperature reached 379°C at JL95. The pH of the venting fluid, the salinity and DO of surrounding deep-sea water are given in Table 1. The JL90, JL94D, and JL94H chimney samples were dominated by the elements of sulfur and iron which composed of pyrite (FeS_2_). Zinc sulfide (ZnS) was a major component of these chimneys. But the JL95 chimney sample was composed of the Bementite and Birnessite according to the results of XRD (Figure S2). The concentration of manganese (3.45 × 10^5^ mg kg^−1^) in JL95 was nearly 5 times more than the iron (6.88 × 10^3^ mg kg^−1^), and much more than for other elements such as sulfur (1.68 × 10^3^ mg kg^−1^) and zinc (1.54 × 10^2^ mg kg^−1^) (Table 1).

### Alpha- and beta- diversity

We targeted the V4-V5 region to characterize the archaeal communities, and V4 region for bacterial communities associated with hydrothermal deposits according to previous report (Flores et al., 2011). In total, we generated average 5.02 × 10^4^ high-quality archaeal sequences per sample (≈410 nt length) and average 2.76 × 10^4^ bacterial sequences (270–275 nt length) per sample for all detected chimneys. Alpha-diversity assessments (Chao1 index and Shannon analysis) provided comparable results showed in Figure S3 and Table 2. Additionally, the Shannon indices indicated that the bacterial diversity (ranged from 6.48 to 10.6) was greater than archaeal diversity (ranged from 4.59 to 6.83) in each sulfide sample (Table 2). The microbial community composition between chimneys samples (beta-diversity) were assessed using OTU-based metrics (Bray-Curtis) to form the neighbor-joining clustering tree with weighted UniFrac. On the whole, the JL90 and JL94D bacterial communities were grouped together, meanwhile the high-temperature vent chimney samples, JL94H and JL95 were scattered in different branch of the tree (Figure S4).

**Table 2:** Diversity estimates from 16S rRNA amplicon libraries: miseq tag sequences.

**Samples**	**Type**	**No. of OTUs**	**Total clean reads^a^**	**Shannon^b^**
JL90A^*^	HT-Active sulfide	665	16,548	4.61 (±0.02)
JL94DA	LT-Active sulfide	1,497	38,787	5.67 (±0.04)
JL94HA	HT-Active sulfide	774	51,649	4.54 (±0.03)
JL95A	HT-Active sulfide	2,034	49,777	6.79 (±0.04)
CH7A	LT-Active sulfide	794	24,720	4.14 (±0.01)
JL90B^*^	HT-Active sulfide	1,563	50,336	6.52 (±0.04)
JL94DB	LT-Active sulfide	1,305	19,932	7.53 (±0.02)
JL94HB	HT-Active sulfide	1,085	22,691	6.76 (±0.03)
JL95B	HT-Active sulfide	4,336	12,203	10.50 (±0.01)
CH7B	LT-Active sulfide	1,034	29,856	5.85 (±0.02)

* *A, archaea; B, bacteria; HT, high-temperature; LT, low-temperature*.

a *Total clean reads after pooling of samples according to Qiime pipeline*.

b *Calculated after subsampling of 10,660 reads for bacterial samples and 13,291 reads for archaeal samples*.

### Taxonomic analysis

Based on the alpha-diversity of each chimney and the beta-diversity among target chimney samples in this study, we attempted to evaluate how the microbial community differences link to possible biogeographic functions.

#### Archaea

**Figure 2:**
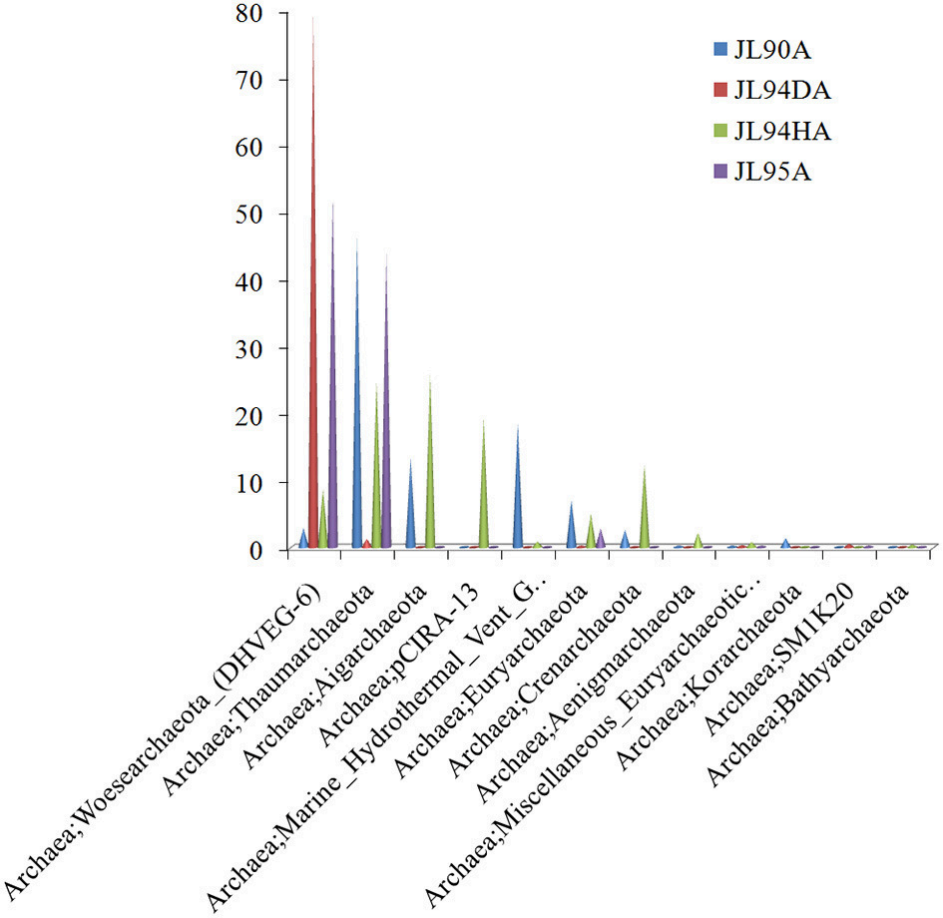
Taxonomic relative abundance of archaeal classes observed of chimney samples for Longqi vent field at SWIR. Bar charts show the Kingdom; Phylum distribution for taxonomically assigned tags that occurred more than 1,000 times.

**Figure 3:**
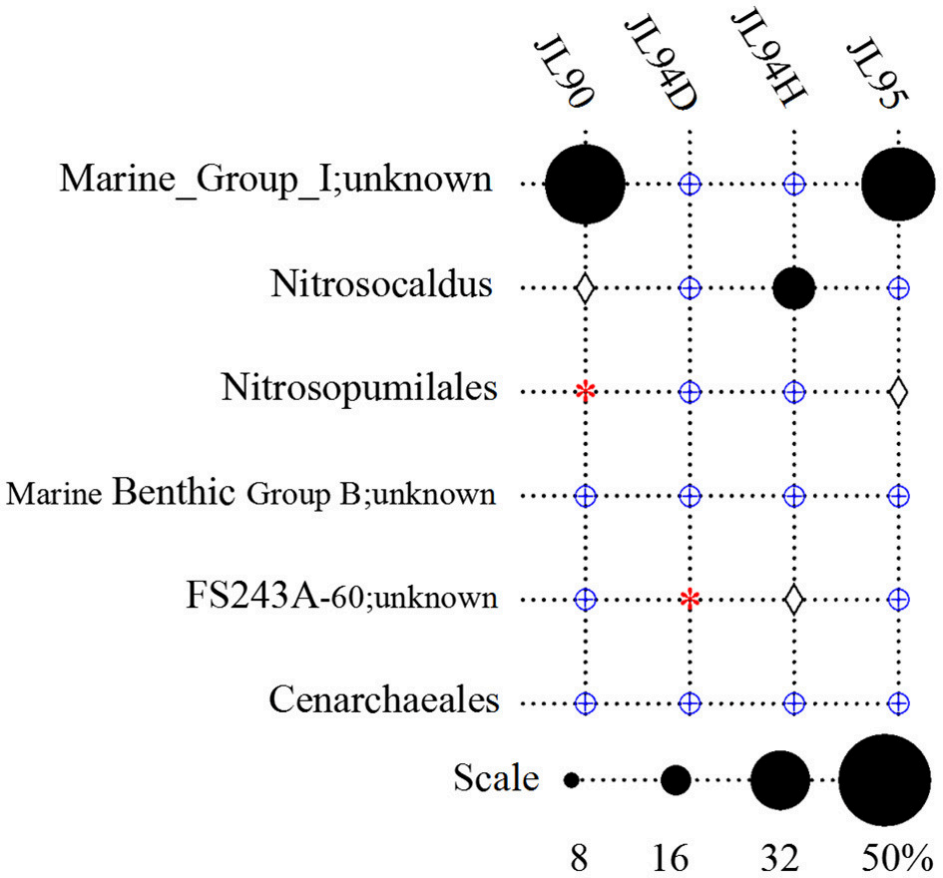
Taxonomic relative abundance of *Thaumarchaeota* genera observed in chimney samples. DHVEG-6, Deep Sea Hydrothermal Vent Group 6. Groups in which no tags were sequenced are indicated with a red asterisk. Groups which were detected below1% are indicated with a blue oplus, between 1 and 2% are indicated with a diamond.

Overall, the four chimney samples from SWIR contained 12 phyla of archaea. The shared communities at different chimney samples were further evaluated. It was found that 11 were shared and only one of the 12 phyla, the candidate phylum SM1K20 was absent in JL90. Over 24% relative abundance of the *Thaumarchaeota* OTUs were found in other libraries except JL94D, which was dominated by Woesearchaeta with 79% relative abundance (Figure 2). *Thaumarchaeota* accounted for the majority of tags in all libraries but also presented different composition pattern among samples. For JL95, the *Thaumarchaeota* was comprised of abundant unclassified MGI and unclassified *Nitrosopumilales*, but for JL90, there was few *Nitrosopumilales* but with high abundance of HWCGIII/Nitrosocaldus OTUs. The HWCGIII/*Nitrosocaldus* was dominant among *Thaumarchaeota* genera of JL94H (Figure 3). Additionally, based on the Venn plot analysis, there are 22 OTUs as the overlap among all the chimney samples, and most of them correspond to the phylum of *Thaumarchaeota* (Figure S6). Other thermophilic lineages shared by most samples include Terrestrial Hot Spring Group (THSCG), Marine Benthic Group E (*Euryarchaeota* MBGE) and *Themoprotei*. The Marine Hydrothermal Vent Group 1 (MHVG-1) was only abundant in the sample JL90. Several novel lineages with no known isolates in culture were also observed. For example, pCIRA-13 and *Bathyarchaeota* were not abundant but found in all samples.

#### Bacteria

**Figure 4:**
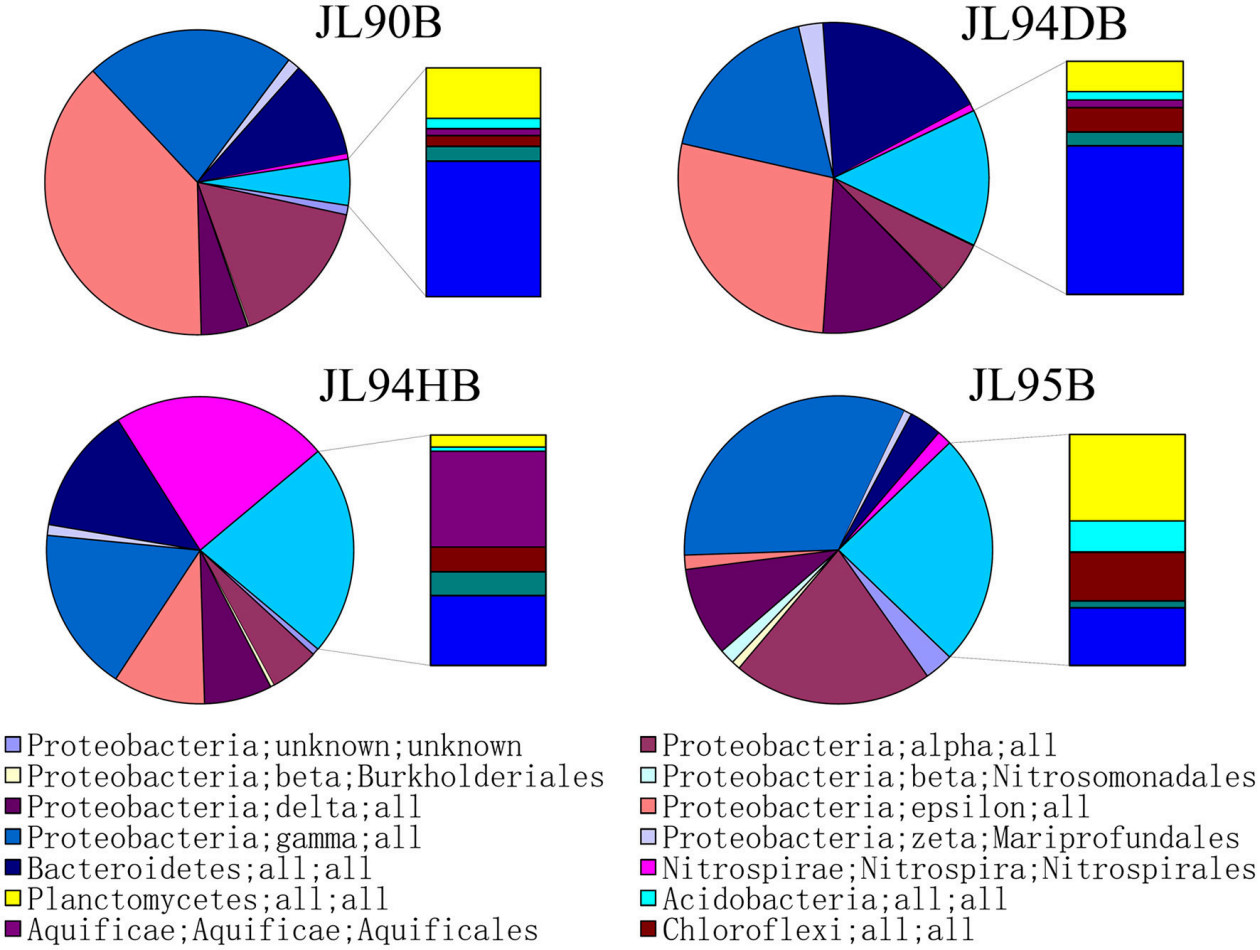
Taxonomic breakdown of bacterial 16S rRNA V4-region tags from each chimney sample. Pie Charts show Phylum; Class; Order distribution (The average relative abundance among samples is over 1%) for taxomomically assigned tags that occurred more than 1,000 times; the remaining tag sequences are grouped into “Other.”

**Figure 5:**
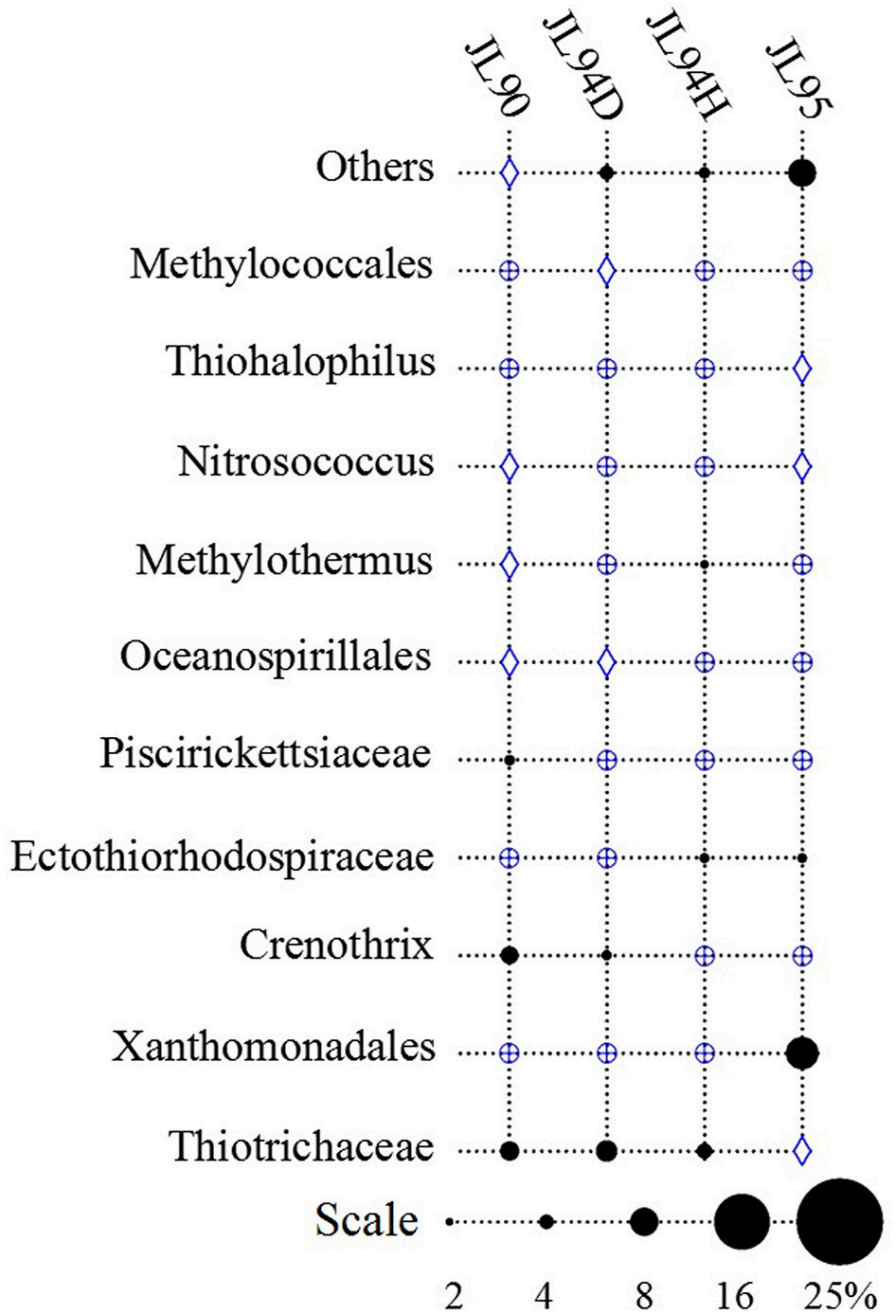
Relative abundance of Gammaproteobacteria taxa observed in each chimney sample for Longqi field at SWIR. “others” represents the less abundant genera of Acinetobacter, Sedimenticola, Marinicella, Colwellia, Arenicellaceae, Marine methylotrophic Group 2, Coxiella, Granulosicoccus, and unclassified Gammaproteobacteria. Groups which were detected below1% are indicated with a blue oplus, between 1 and 2% are indicated with a blue diamond.

**Figure 6:**
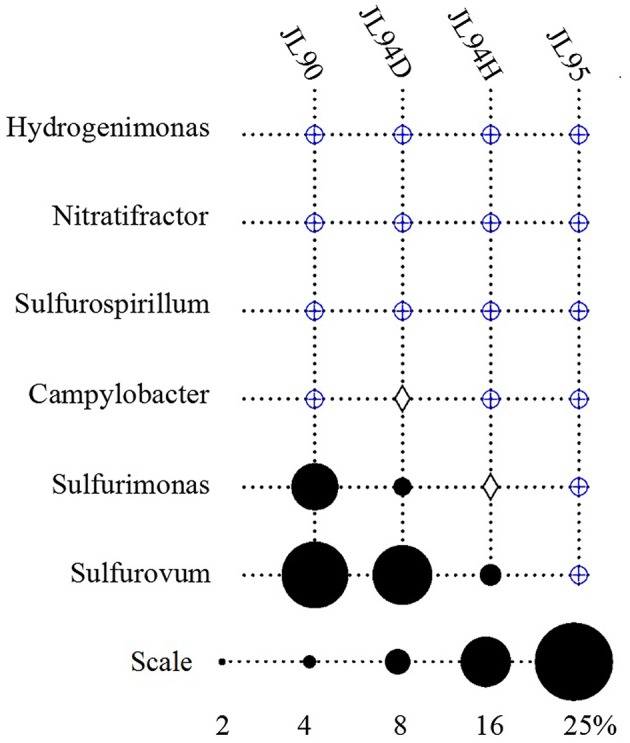
Relative abundance of Epsilonproteobacteria genera observed in each chimney samplefor Longqi field at SWIR. Groups which were detected below1% are indicated with a blue oplus, between 1 and 2% are indicated with a diamond.

The four sulfide samples contained 29 similar phyla except the absence of *Fusobacteria* in JL95. These belonged to several major distinct clusters corresponding to the class of *Gammaproteobacteria, Epsilonproteobacteria, Alphaproteobacteria, Bacteroidetes, Deltaproteobacteria, Nitrospira, Planctomycetes, Actinobacteria, Aquificae, Chloroflexi, Zetaproteobacteria, Deferribacteres*, and *Betaproteobacteria*. Within the four bacterial libraries, JL90B, JL94DB, JL94HB, and JL95B, approximately 22%, 17%, 17% and 30% were classified as *Gammaproteobacteria*, respectively (Figure 4). The *Thiotrichaceae, Xanthomonadales, Crenothrix, Ectothiorhodospiraceae, Piscirickettsiaceae, Oceanospirillales, Methylothermus, Nitrosococcus, Thiohalophilus*, and *Methylococcales* within *Gammaproteobacteria* were observed in all samples. Numerous unclassified *Gammaproteobacteria* was also recovered from all samples (Figure 5). Additionally, there was a decrease of *Epsilonproteobacteria* in libraries along with the increase of temperature of chimney fluids. The composition of *Epsilonproteobacteria* was sharply shifted among different chimney samples range from 37.8% in JL90 and 26.6% in JL94D to 1.4% in JL95. Within the *Epsilonproteobacteria*, the genus of *Sulfurovum, Sulfurimonas, Campylobacer, Sulfurospirillum Nitratifractor*, and *Hydrogenimonas* were detected in all chimney samples with quite different relative abundance (Figure 6).

## Discussion

### Methodological considerations

In this study, high-throughput, DNA-based analysis of environmental samples has been applied to investigate the microbial communities of chimney samples collected from Longqi hydrothermal field. Recent studies have found archaeal DNA was poorly recovered from lower temperature, diffuse flow vents or inactive chimneys (Bourbonnais et al., 2012; Sylvan et al., 2012; Gulmann et al., 2015) and speculated that it was due to low low abundance or primer mismatch. In this study, we presented the archaeal community analysis of chimney samples including low-temperature deep-sea hydrothermal vent from Longqi field at SWIR using high-throughput Miseq sequencing. A total of 110 473 and 200 630 amplicons of bacterial and archaeal 16S rRNA genes, respectively, have been obtained, suggesting that the methods appeared to be efficient. Deep sequencing of archaea and bacteria from those chimneys revealed thousands of bacterial and archaeal lineages, the majority of which appeared in very low abundance just as presented in the previous studies (Sogin et al., 2006; Huber et al., 2007).

### Microbial diversity and connection with regional geochemical parameters

According to the Venn diagrams showing (Figure S6), JL94D and JL94H shared the same sampling location and also the highest percent of bacterial OTUs, 9.6%, followed by the overlap between JL90 and JL94D, 7.7%, JL90, and JL94H, 7.6%. Besides, the bacterial OTUs overlap between JL95 and JL94 or JL90 about or below 6.2%. The species composition overlap reflected the microbial resemblance among the four chimneys at Longqi field on SWIR.

Microbial community structures were clearly correlated to the environmental parameters, and among all the considered parameters the *in situ* temperature was the most influential one. Previous studies demonstrated that the biogeographical patterns of microbial communities were shaped in part by local fluid geochemistry in active hydrothermal vent chimneys (Flores et al., 2011), mineralogy on inactive seafloor sulfide deposits (Toner et al., 2013) and geological processes, such as eruption, on diffuse-flow vents (Gulmann et al., 2015). To evaluate the effects of linear correlation between environmental factors and microbial communities, redundancy analysis (RDA) is used in this study (Table S1). Compared to the other high-temperature vents and low-temperature vent of JL94D, the RDA analysis also confirmed the separation of JL95 from other chimneys, correlated with a significant decrease of sulfur, Fe, and Zn content, with the increase of temperature of fluid and manganese content of chimney (Figure S5). But there was no genus which was found to have a significant linear correspondence with any environmental factor. Therefore, we assumed that regional geochemical condition might have affected the structure and function of the microecosystem in the SWIR region.

### Inferred microbial metabolic potentials in energy metabolism

Potential energy sources for deep-sea vent chemoautotrophy include reduced sulfur compounds, molecular hydrogen, reduced metals and ammonium. Classic sulfur-oxidizing bacteria have been detected as the dominant families, suggesting a strong sulfur-metabolizing potential in all tested chimney samples (Table 3). Many members within *Epsilon*- and *Gammaproteobacteria* are known to be chemoautotrophs utilizing inorganic sulfur as election donor to gain energy (Nakagawa et al., 2005; Yamamoto and Takai, 2011; Anderson et al., 2015). The deep-sea chemoautotrophic *Gammaproteobacteria*, possess two different sulfur-oxidization pathways including the reverse sulfate reduction and the Sox multienzyme system without SoxCD, and strictly require co-existence of reduced sulfur compounds and O_2_ (Yamamoto and Takai, 2011). In this study, *Gammaproteobacteria* dominated in all detected chimney samples with the sulfide-oxidizing bacterium within the genera of *Thiotrichaceae, Ectothiorhodospiraceae, Thiohalophilus*, and *Piscirickettsiaceae* (Figure 4). It seemed to indicate that both the reduced sulfur compounds and O_2_ were steadily supplied into the chimney habitats. *Epsilonproteobacteria* were known to play a significant role in carbon, nitrogen and sulfur cycling and had consistently shown to be the most numerically abundant bacteria in sediment (López-García et al., 2003), hydrothermal fluids (Huber et al., 2010), hydrothermal plumes (Nakagawa et al., 2005), and vent chimneys (Campbell et al., 2001; Opatkiewicz et al., 2009; Dahle et al., 2013). Based on our data, Epsilonproteobacterial amplicons ranged from 1.3% at high-temperature vent chimneys to 37.6% for diffusive vent chimneys. Certain sequences dominated and closely related to the known chemosynthetic, sulfur-oxidizing genera *Sulfurovum* and *Sulfurimonas*. Similar communities were also found in cool, diffusive flow at Axial Seamount on the Juan de Fuca Ridge (Akerman et al., 2013) and the biofilms growing on the chimney walls at the Loki's Castle vent field (Dahle et al., 2013). The most abundant genera of *Sulfurovum* and *Sulfurimonas* within *Epsilonproteobacteria* were recovered from all active chimneys in our study, and also recovered from inactive sulfides in the EPR (Sylvan et al., 2012). It is possible that these groups represent the widely distributed species at active sulfides and the survivable relict populations at inactive chimneys by oxidizing sulfide minerals.

**Table 3:** Potential ecological function of tag sequences for which obvious metabolisms can be inferred.^*^

**Function**	**Bacterial/Archaeal taxonomy**	**Relative abundance (%)**			
		**JL90**	**JL94D**	**JL94H**	**JL95**
**BACTERIA**					
S oxidation	*Aquificae; Aquificales; Aquificaceae; Hydrogenivirga*	0.016	0.105	1.780	0.000
	*Epsilonproteobacteria; Campylobacterales; Helicobacteraceae*	36.326	24.724	8.633	1.246
	*Gammaproteobacteria; unknown; unknown; Thiohalophilus*	0.193	0.040	0.855	1.065
	*Gammaproteobacteria; Thiotrichales; Thiotrichaceae*	5.360	5.955	4.160	1.950
	*Gammaproteobacteria; Thiotrichales; Piscirickettsiaceae*	2.908	0.642	0.282	0.295
	*Gammaproteobacteria; Chromatiales; Ectothiorhodospiraceae*	0.971	0.191	2.728	2.663
Sulfate reduction	*Nitrospira; Nitrospirales; Nitrospiraceae; Thermodesulfovibrio*	0.429	0.657	22.053	0.246
	*Deltaproteobacteria; Desulfarculales; Desulfarculaceae; Desulfatiglans*	0.006	0.557	0.154	0.008
	*Deltaproteobacteria; Desulfobacterales; Desulfobacteraceae*	0.034	1.786	0.604	0.016
	*Deltaproteobacteria; Desulfobacterales; Desulfobulbaceae*	3.028	5.398	3.790	0.131
	*Epsilonproteobacteria; Campylobacterales; Campylobacteraceae; Sulfurospirillum*	0.360	0.627	0.194	0.008
	*Thermodesulfobacteria; Thermodesulfobacteriales; Thermodesulfobacteriaceae; Thermosulfurimonas*	0.022	0.060	1.044	0.008
**Sum of S oxidation and sulfate reduction**		49.653	40.742	46.277	7.636
Ammonia oxidation	*Gammaproteobacteria; Chromatiales; Chromatiaceae; Nitrosococcus*	1.196	0.115	0.375	1.868
Nitrite oxidation	*Nitrospira; Nitrospirales; Nitrospiraceae; Nitrospira*	0.002	0.005	0.026	0.992
Nitrate reduction	*Epsilonproteobacteria; Nautiliales; Nautiliaceae; Nitratifractor*	0.489	0.105	0.062	0.057
Nitrification	*Betaproteobacteria; Nitrosomonadales; Nitrosomonadaceae; Nitrosomonas*	0.002	0.010	0.004	1.418
N fixation	*Alphaproteobacteria; Rhizobiales*	5.739	0.562	0.450	2.622
**Sum of ammonia, nitrite oxidation, nitrification and N fixation**		7.428	0.797	0.917	6.957
H oxidation	*Aquificae; Aquificales; Aquificaceae; Hydrogenobacter*	0.028	0.080	1.908	0.016
	*Aquificae; Aquificales; Hydrogenothermaceae; Persephonell*	0.072	0.261	4.958	0.033
	*Epsilonproteobacteria; Campylobacterales; Hydrogenimonaceae; Hydrogenimonas*	0.290	0.025	0.198	0.008
**Sum of H Oxidation**		0.39	0.366	7.064	0.057
CH4 oxidation	*Gammaproteobacteria; Methylococcales; Methylococcaceae; Methylothermus*	1.122	0.161	2.318	0.057
Fe(II) oxidation	*Zetaproteobacteria; Mariprofundales; Mariprofundaceae; Mariprofundus*	1.295	2.468	1.084	0.705
Fe(III) reduction	*Deltaproteobacteria; Desulfuromonadales; Desulfuromonadaceae; Desulfuromusa*	0.238	1.801	0.591	0.041
**Sum of Fe(II) oxidation and Fe(III) reduction**		2.655	4.43	3.993	0.803
Mn oxidation	*Alphaproteobacteria; Rhodobacterales; Rhodobacteraceae; Roseobacter*	0.068	1.234	0.353	0.049
Total bacteria		60.194	47.569	58.604	15.502
**ACHAEA**					
Sulfate reduction	*Crenarchaeota; Thermoprotei; Desulfurococcales*	0.538	0.003	7.460	0.014
	*Crenarchaeota; Thermoprotei; Thermoproteales*	0.326	0.003	1.111	0.004
Ammonia oxidation	*Thaumarchaeota*	45.667	1.062	24.636	42.745
Total archaea		46.531	1.068	33.207	42.763

* *The relative abundance in sequencing library is for each sample's Miseq data set. Taxa are designated by class (phylum for Crenarchaeota and Thaumarchaeota), order, family, and genus*.

Ammonia oxidation is the first step of nitrification, in which ammonia is first oxidized to nitrite by ammonia-oxidizing bacteria and/or archaea (AOB or AOA), then subsequently to nitrate by nitrite-oxidizing bacteria (NOB). The *Thaumarchaeota* had rapidly gained much attention after the discovery that some of them have been able to oxidize ammonia aerobically, providing the first example of nitrification in the Archaea and therefore extending the range of microorganisms capable of this important metabolism, which was previously thought to be restricted to a few proteobacterial lineages (Könneke et al., 2005). Therefore, we deduced that *Thaumarchaeota* might be the major Ammonia Oxidizing Archaea (AOA) among the recovered microorganisms at high-temperature vent chimneys JL94H and JL95 with relative abundance over 40%, also for JL90 with ~24% (Figure 2). Besides, the Ammonia Oxidizing bacteria (AOB) within *Nitrosococcus* were recovered and represented the abundant genus (over 1%) in JL90 and JL95. Related genus of nitrifier, the *Nitrospira*, and *Nitrosomonas*, were found in all samples and with highest abundance in JL95 (Table 3). Recently, the completely nitrifying bacterium from the genus *Nitrospira* was reported (Daims et al., 2015), indicating that the globally distributed nitrite oxidizers fundamentally changed the picture of nitrification and might act as key microbial communities involved in nitrogen-cycling on the high-temperature chimney JL95 and other samples at Longqi hydrothermal field.

The reduced metals (Fe, Mn, Cu, etc.,) are the endmembers in vent fluids and potential energy sources for deep-sea vent chemoautotrophs. Fe(II) is a common and often the most dominant metal. Microaerophilic Fe-oxidizing microorganisms (FeOM) colonize gradients of Fe(II) and oxygen, taking advantage of the available chemical energy. Vast communities of FeOM proliferate at deep sea hydrothermal vents, forming mineralized mats (Chan et al., 2013). The “zetaproteobacterium” *Mariprofundus ferooxydans* (Emerson and Moyer, 2002) from Loihi Seamount, and several Alpha- and *Gammaproteobacteria* strains are reported as the chemoautotrophic Fe(II) oxidizers described from deep-sea vents (Edwards et al., 2003). *Mariprofundus* is the sole member of the class *Zetaproteobacteria* in the phylum *Proteobacteria*. Several cultured members (JV-1, PV-1) of Fe-oxidizing *Mariprofundus* were isolated from deep-sea hydrothermal fields (Singer et al., 2011; Makita et al., 2017). The genus of *Mariprofundus* were observed as only abundant FeOM group (relative abundance over 1%) in the chimney samples JL90, JL94D, and JL94H, except in JL95 with 0.705 (Table 3), indicating that Fe-oxidizing bacteria within *Mariprofundus* were common at the Longqi field and probably played a major role in Fe oxidation. Mn(II) oxidation mediated by heterotrophic Bacillus species in Guaymas Basin hydrothermal plumes as reported previously (Dick et al., 2006). Those species were not recovered in any of the chimney samples collected from Longqi field at SWIR, but the genus of *Roseobacter* in *Alphaproteobacteria* were observed and inferred as the Mn(II) oxidizer.

Hydrogen has also been shown to be an important energy source in vent fluids at the Logatchev and Rainbow fields on the Mid-Atlantic Ridge (Takai et al., 2006). Chemoautotrophs with the ability to derive energy from H_2_ oxidation have been isolated from various deep-sea hydrothermal fields, including *Aquificales, Epsilonproteobacteria, Desulfurococcales, Methanococcales, Thermodesulfobacteriales*, and *Deferribacterales* (Nakagawa and Takai, 2008). Analysis of tag sequences revealed members of the genera *Hydrogenobactera* and *Persephonell* within the *Aquificae* and *Hydrogenimonas* in the *Epsilonproteobacteria* (Table 3). These groups are likely H_2_-oxidizing bacteria.

### Trans-regional distribution pattern in microbial communities of hydrothermal vents

**Figure 7:**
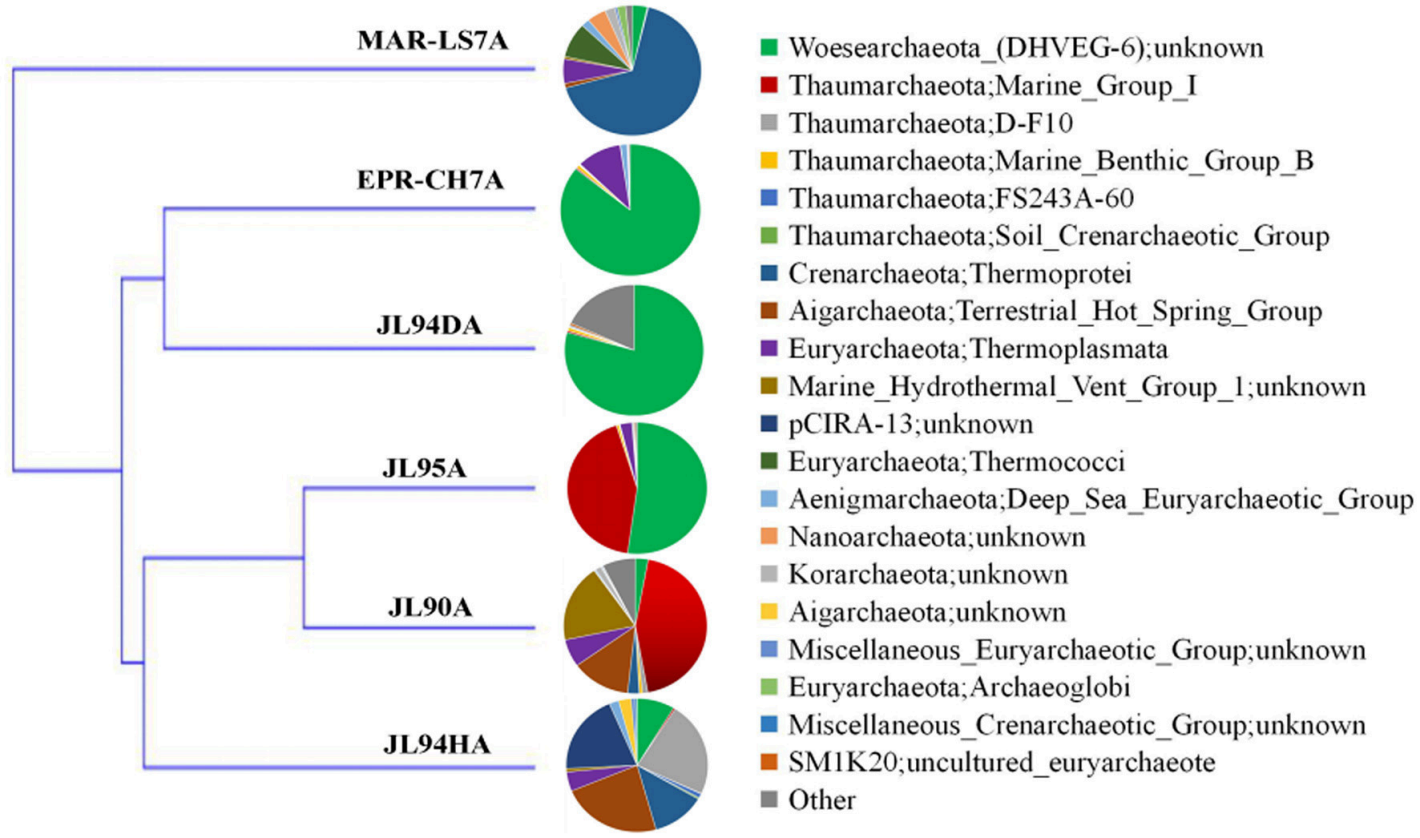
Clustering analysis tree of the archaeal community structure of chimneys from SWIR, EPR and MAR. LS7 = Lucky Strike. Lucky Strike vent field located at MAR (Flores et al., 2011); CH7 = chimney sample from EPR, this study.

**Figure 8:**
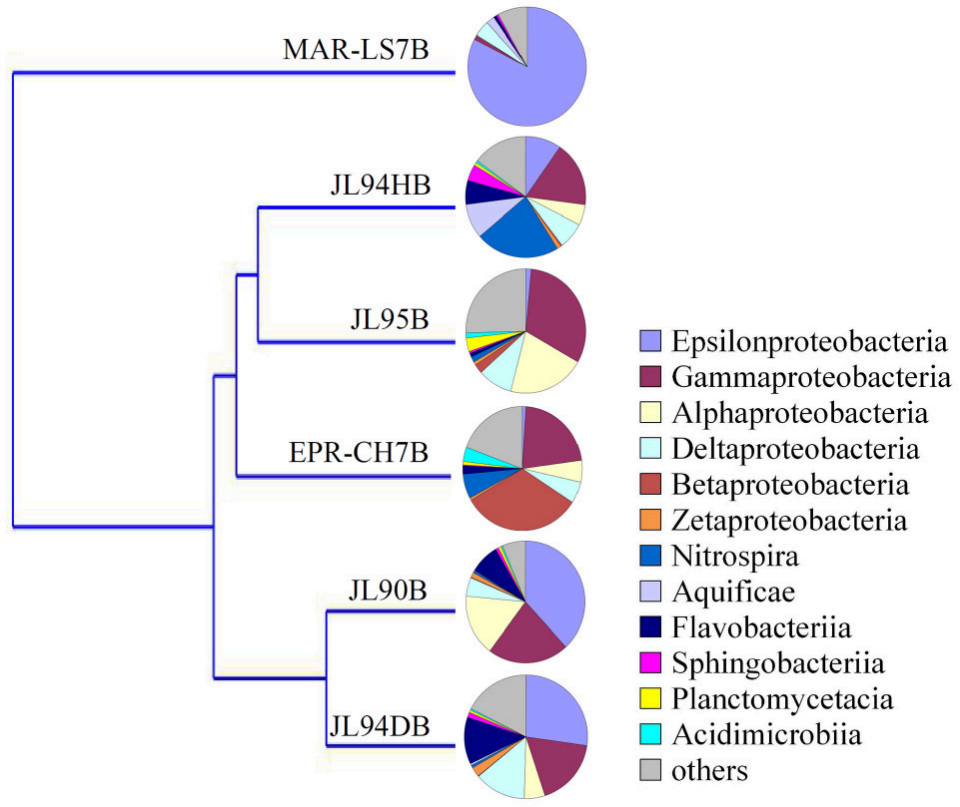
Clustering analysis tree of the bacterial community structure of chimneys from SWIR, EPR and MAR. LS7 = Lucky Strike. Lucky Strike vent field located at MAR (Flores et al., 2011); CH7 = Chimney sample from EPR, this study.

To evaluate the effects of geological and geochemical characteristic on microbial communities on the surface of active chimneys in Longqi field at SWIR, we compared the bacterial and archaeal distribution pattern with habitats of active chimney both from slow-spreading ridge of MAR and fast-spreading ridge of EPR. Overall, the microbial composition on active chimneys recovered by tag sequencing at SWIR in our libraries was different from hydrothermal vents at EPR and MAR by using cluster analysis (Figures 7, 8). The results showed that all archaeal communities from chimneys at SWIR and EPR were clustered into different branches from the high-temperature vent chimney in MAR. On the other hand, the high-temperature vent chimney LS7 from MAR was highly dominated by Epsilonproterobacteria, which quite different from the bacterial composition of other active chimneys from SWIR and EPR, might lead to be clustered into a separate branch. The bacterial communities of JL95 and CH7 from EPR were surprisingly clustered in to a group with limited *Epsilonproteobacteria*, but with dominant Gammaproteobacterial sulfur oxidizers. The order *Xanthomonadales* within *Gammaproteobacteria* was only dominantin JL95, which was also observed in the microbial community of deep-sea sediment (Li et al., 2015). Within archaeal communities, the culturable genus of *Pyrococcus* and *Thermococcus* were recovered frequently in molecular environmental surveys at hydrothermal vent (Edgcomb et al., 2007; Flores et al., 2011; Li et al., 2014), but were absent in our archaeal tag sequences, which might be because of the distinct geographic locations and geochemical conditions related.

## Conclusions

This study reported the distribution and diversity of the prokaryotic communities on the surface of chimneys collected from Longqi field the SWIR. The 16S rRNA gene analysis suggested that bacterial communities were highly diversified among all the detected samples. Compared to bacteria, the lower diversity of archaeal phylotypes agreed with other molecular surveys indicating that marine hydrothermal vent archaeal diversity is relatively limited (Huber et al., 2007). Phylotypes, belonging to *Gammaproteobacteria* and *Epsilonproteobacteria*, appeared to be diverse and abundant in most of samples. In contrast to the broad taxonomic coverage of bacterial community, archeal 16S rRNA gene sequences were predominated by the *Thaumarchaeota* and *Woesearchaeota*. Based on the functional analysis of bacteria and archaeal communities, sulfur-oxidation and reduction may be important energy metabolism pathways in low- and high-temperature vent chimneys with high abundance of SOB within *Gammaproteobacteria* and *Epsilonproteobacteria*. Meanwhile, ammonia oxidation may be another major pathway to provide energy for microbial ecology system on high temperature active chimneys. This paper described the results of a molecular phylogenetic analysis of chimneys collected from the Longqi field at ultra-spreading South West Indian ridge by using high through sequencing method. Our results provided more details to characterize the microbial roles in ecologic and minerogenic processes at the SWIR, especially in the S and N cycling.

## Author contributions

HJ and YZ: did the sampling during cruise; XX and JD: designed experiments; JD and HW: carried out experiments; JD, HW, and HL: analyzed experimental results; HL: assisted with Illumina sequencing. JD and YZ: wrote the manuscript.

### Conflict of interest statement

The authors declare that the research was conducted in the absence of any commercial or financial relationships that could be construed as a potential conflict of interest.
