# Supplementary figures and images for: Microbial Community Structure of Deep-sea Hydrothermal Vents on the Ultraslow Spreading Southwest Indian Ridge
Source: Front Microbiol. 2017 Jun 13;8:1012. doi: 10.3389/fmicb.2017.01012 (PMC5468387; doi:10.3389/fmicb.2017.01012)

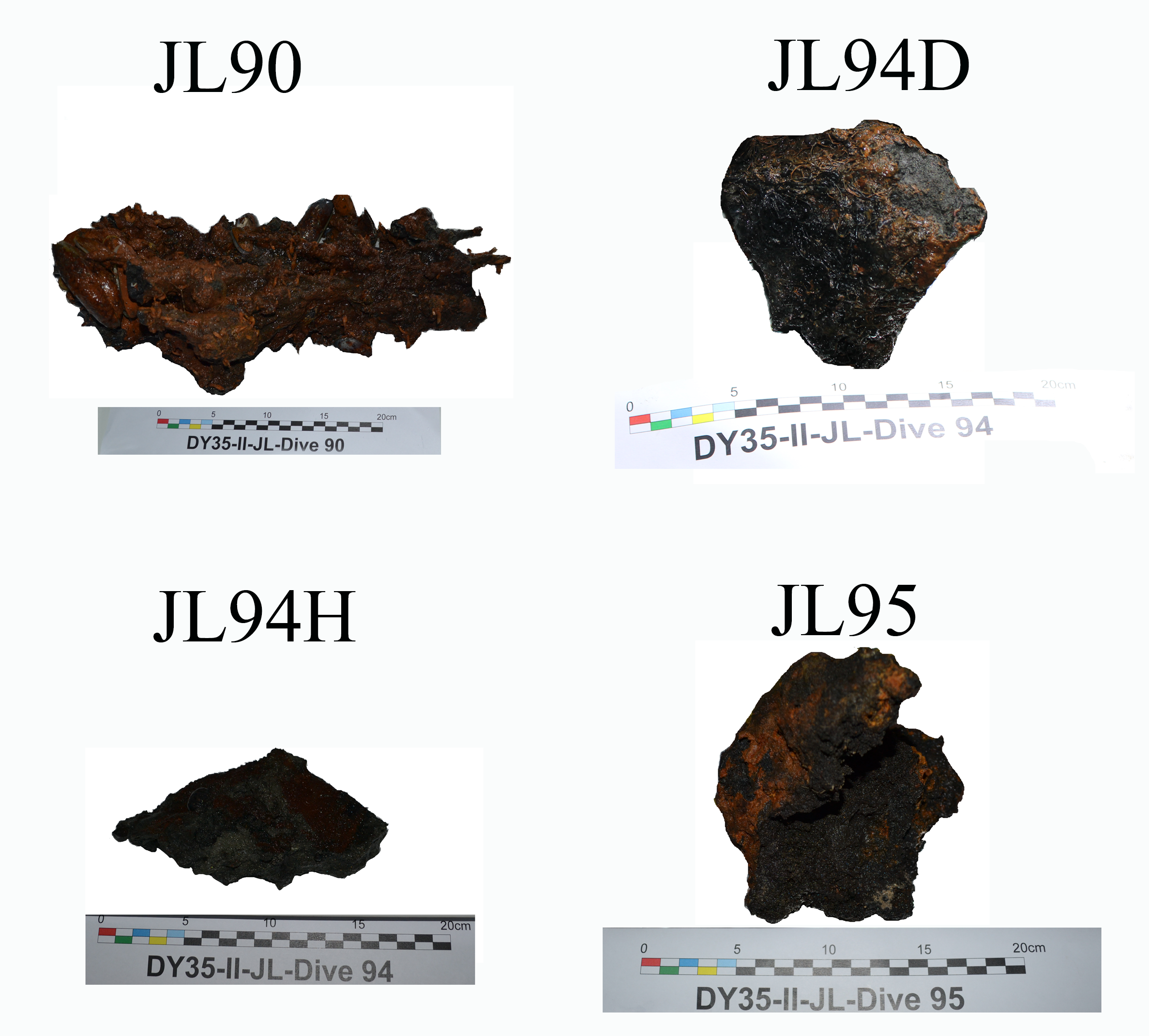

Supplement: Supplementary file 1 [file Image1.TIF]

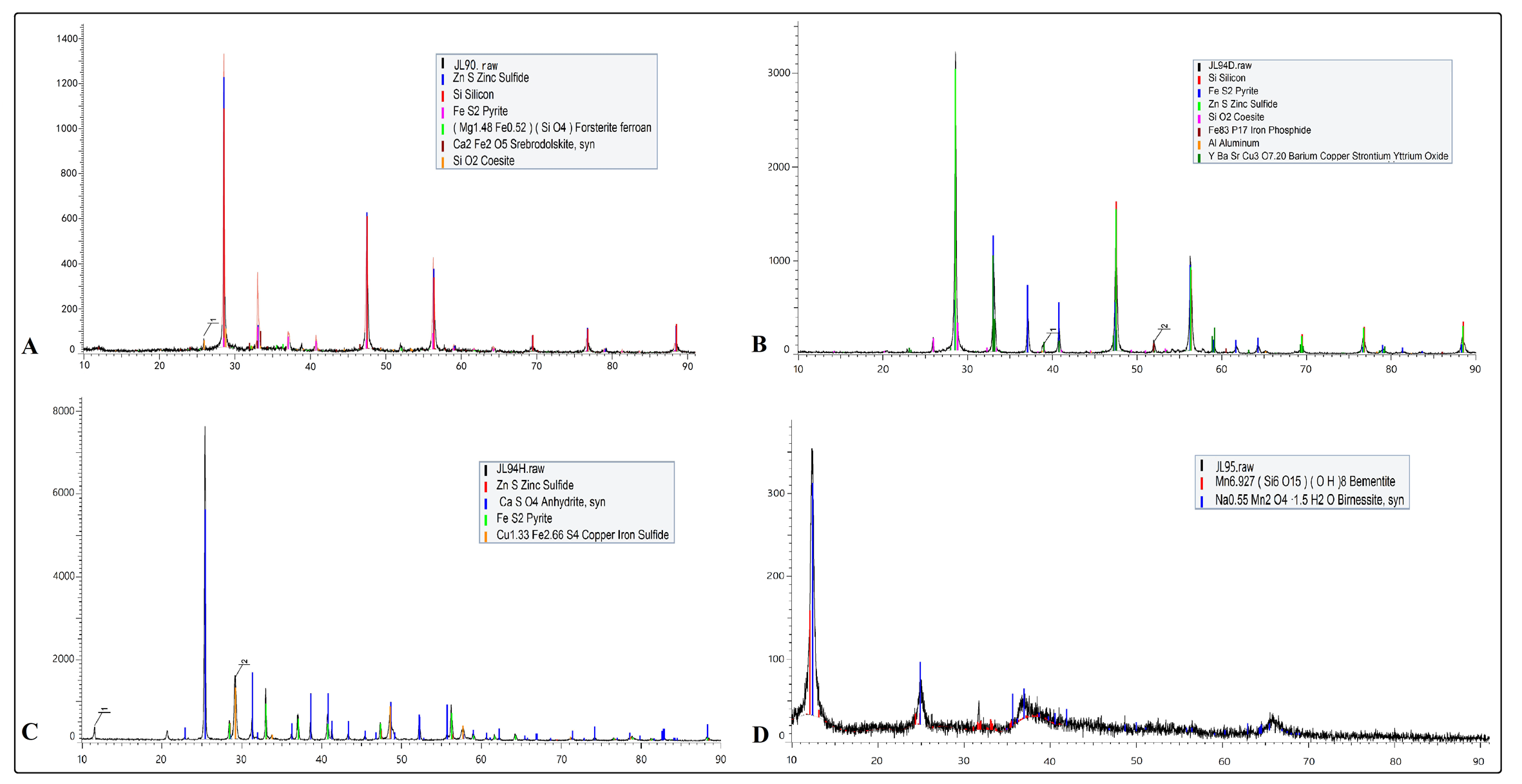

Supplement: Supplementary file 2 [file Image2.TIF]

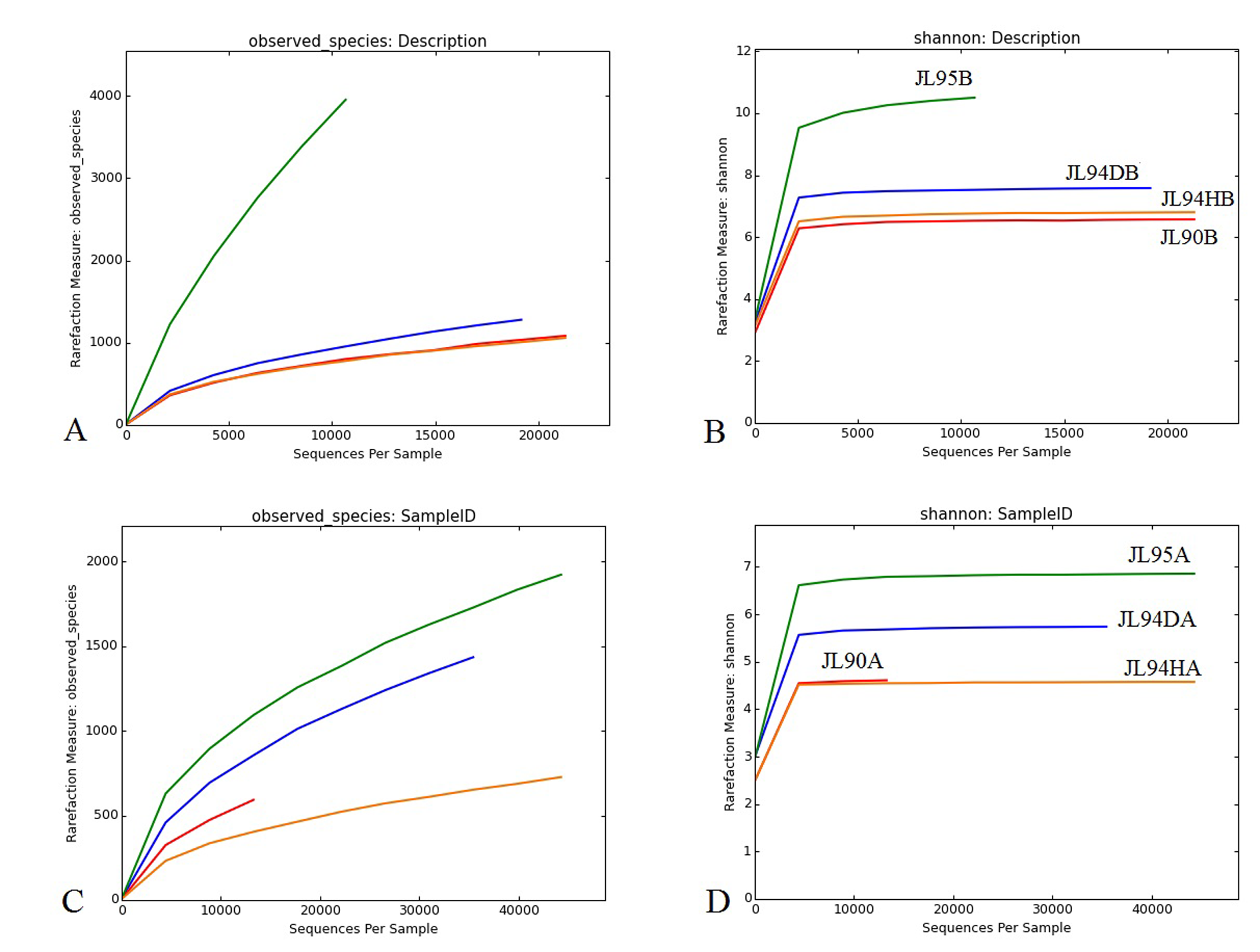

Supplement: Supplementary file 3 [file Image3.TIF]

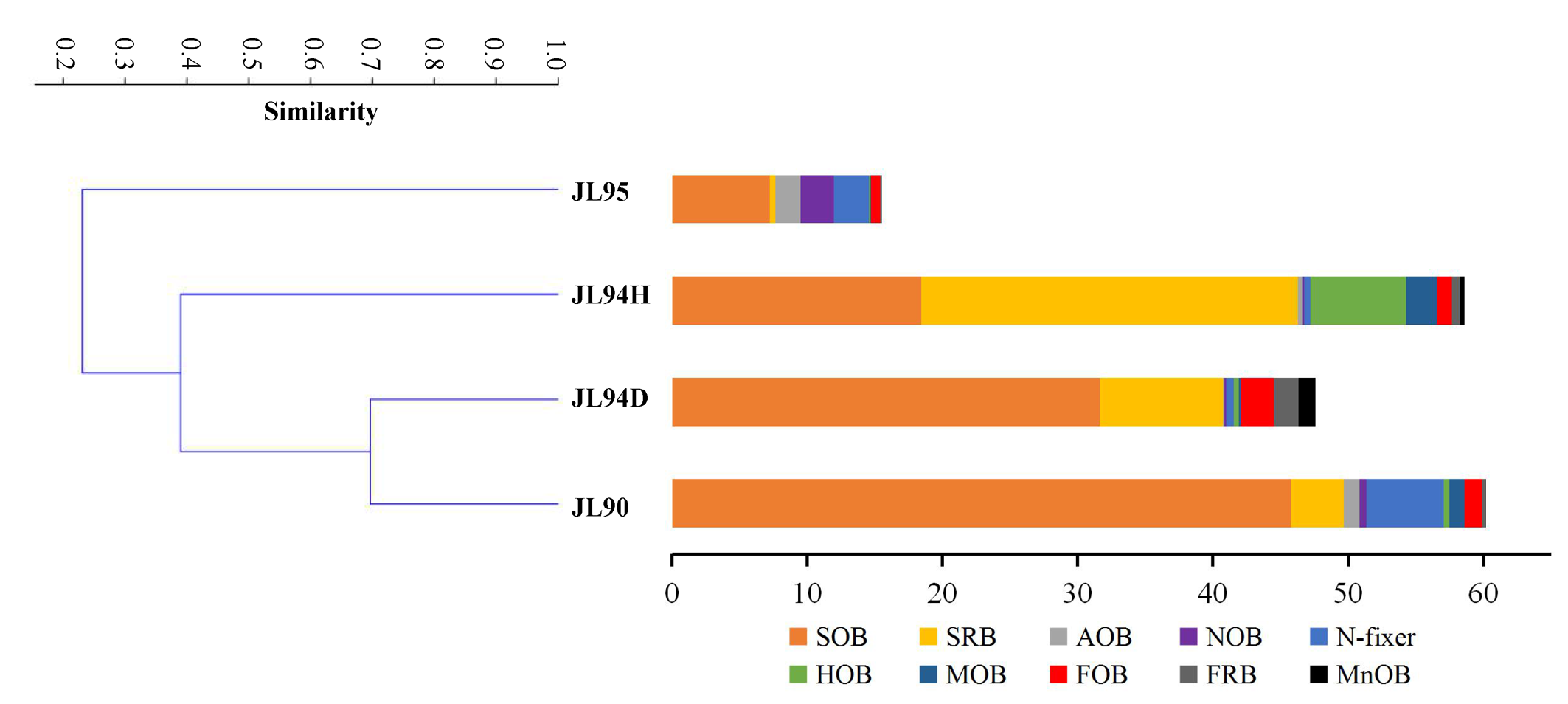

Supplement: Supplementary file 4 [file Image4.TIF]

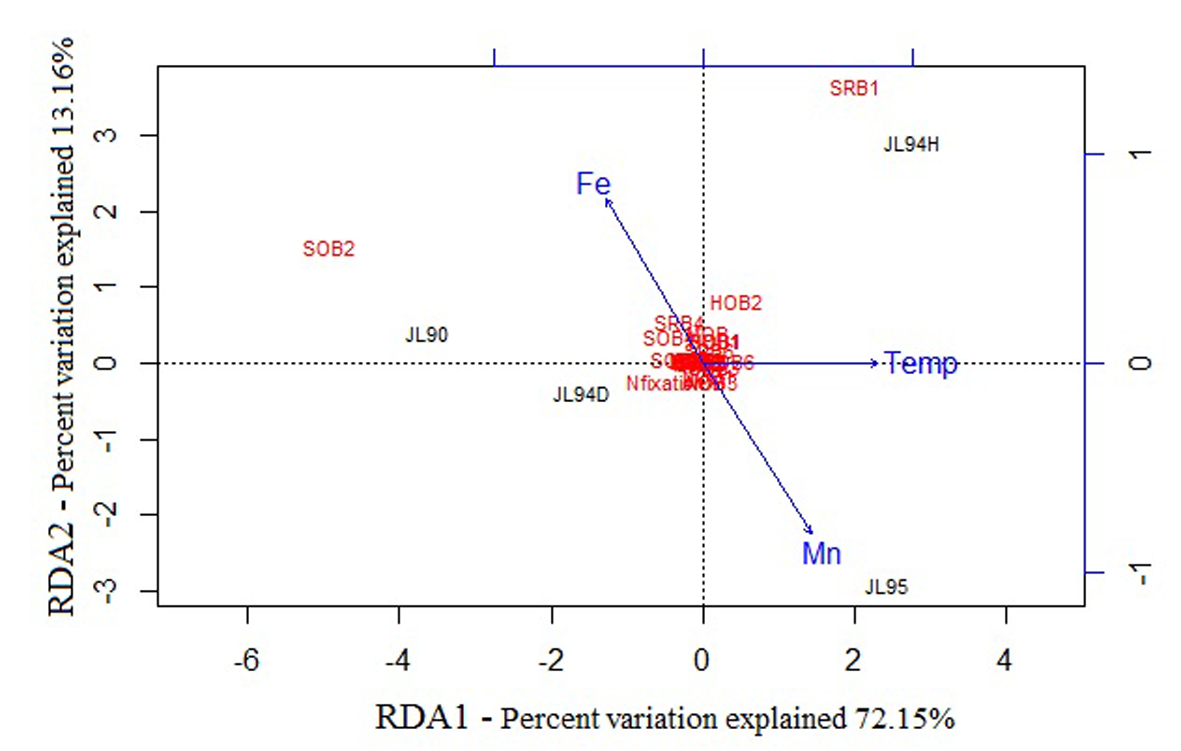

Supplement: Supplementary file 5 [file Image5.TIF]

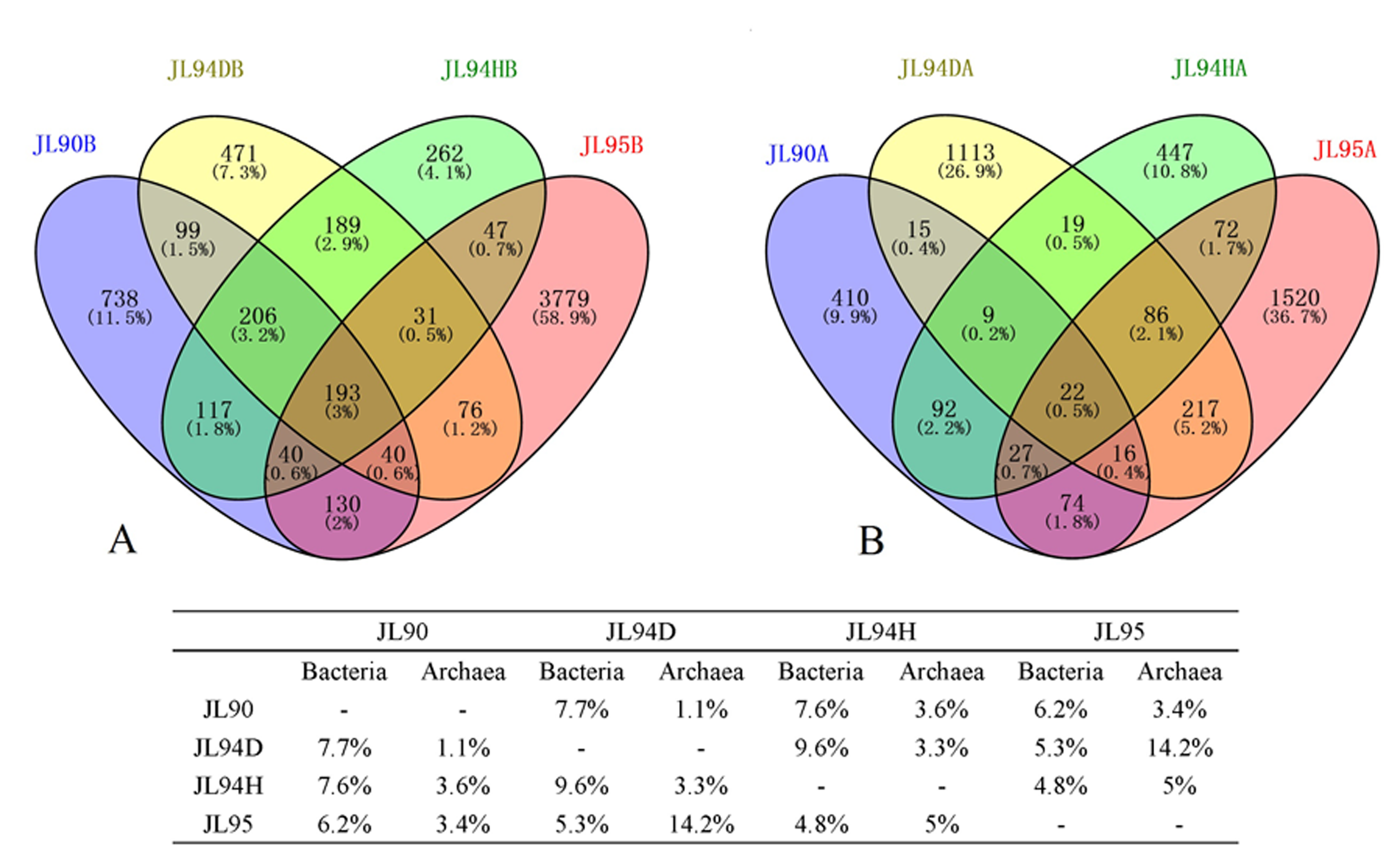

Supplement: Supplementary file 6 [file Image6.TIF]
