# Supplementary material for: Microbial Community Structure of Deep-sea Hydrothermal Vents on the Ultraslow Spreading Southwest Indian Ridge
Source: Front Microbiol. 2017 Jun 13;8:1012. doi: 10.3389/fmicb.2017.01012 (PMC5468387; doi:10.3389/fmicb.2017.01012)
Supplement: Supplementary file 7 [file Table1.DOCX]

Table 1 Concentrations of Elements in chimneys’ deposits and physicochemical characteristics of

their hydrothermal fluids at the Longqi field on SWIR

| Chimneys | JL90 | JL94D | JL94H | JL95 | Range |
| --- | --- | --- | --- | --- | --- |
| Element(mg/kg) | | | | | |
| Fe | 261,600 | 305,900 | 286,500 | 68,780 |  |
| S | 300,200 | 394,400 | 302,400 | 1,682 |  |
| Zn | 211,200 | 233,100 | 2,938 | 154 |  |
| Mn | 3,786 | 33 | 14 | 343,800 |  |
| Mg | 20,960 | 24 | 324 | 13,720 |  |
| Ca | 9,268 | 112 | 46,000 | 13,140 |  |
| Pb | 1,417 | 703 | 57 | 41 |  |
| Cu | 806.8 | 541 | 67,330 | 42 |  |
| Al | 661.6 | 5,680 | 141 | 429 |  |
| Depth (m) | 2746 | 2768 | 2778 | 2775 |  |
| Location | 49.6501525°E, 37.7832506°S | 49.6487677°E, 37.7832151°S | 49.6487677°E, 37.7832151°S | 49.6477092°E， 37.7799720°S |  |
| Fluid | | | | | |
| Max. Temp. (℃) | 145 | 13.3 | 362 | 379 | 13.3-379 |
| pH | 4.85 | - | - | 3.42 | 3.21-4.85 |
| Salinity | 4.0 | - | - | 4.0 | 3.7-4.5 |
| DO (mg/L) | 24.7 | - | - | 14.3 | 0.5-24.7 |

Table 2 Diversity estimates from 16S rRNA amplicon libraries: miseq tag sequences

| Samples | type | No. of OTUs | Total clean reads^a^ | Shannon^b^ |
| --- | --- | --- | --- | --- |
| JL90A* | HT-Active sulfide | 665 | 16548 | 4.61 (±0.02) |
| JL94DA | LT-Active sulfide | 1497 | 38787 | 5.67 (±0.04) |
| JL94HA | HT-Active sulfide | 774 | 51649 | 4.54 (±0.03) |
| JL95A | HT-Active sulfide | 2034 | 49777 | 6.79 (±0.04) |
| CH7A | LT-Active sulfide | 794 | 24720 | 4.14 (±0.01) |
| JL90B* | HT-Active sulfide | 1563 | 50336 | 6.52 (±0.04) |
| JL94DB | LT-Active sulfide | 1305 | 19932 | 7.53 (±0.02) |
| JL94HB | HT-Active sulfide | 1085 | 22691 | 6.76 (±0.03) |
| JL95B | HT-Active sulfide | 4336 | 12203 | 10.50 (±0.01) |
| CH7B | LT-Active sulfide | 1034 | 29856 | 5.85 (±0.02) |

“*” A: archaea; B: bacteria; HT: high-temperature; LT: low-temperature.

^a^ Total clean reads after pooling of samples according to Qiime pipeline

^b^ Calculated after subsampling of 10,660 reads for bacterial samples a 13,291 reads for

archaeal samples.

Table 3 Potential ecological function of tag sequences for which obvious metabolisms can be inferred*

| Potential ecological function | Bacterial/ Archaeal Taxonomy | | Relative abundance (%) | | | |
| --- | --- | --- | --- | --- | --- | --- |
|  |  | | JL90 | JL94D | JL94H | JL95 |
| **Bacteria** |  | |  |  |  |  |
| S oxidation | *Aquificae;Aquificales;Aquificaceae;Hydrogenivirga* | | 0.016 | 0.105 | 1.780 | 0.000 |
|  | *Epsilonproteobacteria;Campylobacterales;Helicobacteraceae* | | 36.326 | 24.724 | 8.633 | 1.246 |
|  | *Gammaproteobacteria;unknown;unknown;Thiohalophilus* | | 0.193 | 0.040 | 0.855 | 1.065 |
|  | *Gammaproteobacteria;Thiotrichales;Thiotrichaceae* | | 5.360 | 5.955 | 4.160 | 1.950 |
|  | *Gammaproteobacteria;Thiotrichales;Piscirickettsiaceae* | | 2.908 | 0.642 | 0.282 | 0.295 |
|  | *Gammaproteobacteria;Chromatiales;Ectothiorhodospiraceae* | | 0.971 | 0.191 | 2.728 | 2.663 |
| Sulfate reduction | *Nitrospira;Nitrospirales;Nitrospiraceae;Thermodesulfovibrio* | | 0.429 | 0.657 | 22.053 | 0.246 |
|  | *Deltaproteobacteria;Desulfarculales;Desulfarculaceae;Desulfatiglans* | | 0.006 | 0.557 | 0.154 | 0.008 |
|  | *Deltaproteobacteria;Desulfobacterales;Desulfobacteraceae* | | 0.034 | 1.786 | 0.604 | 0.016 |
|  | *Deltaproteobacteria;Desulfobacterales;Desulfobulbaceae* | | 3.028 | 5.398 | 3.790 | 0.131 |
|  | *Epsilonproteobacteria;Campylobacterales;Campylobacteraceae;Sulfurospirillum* | | 0.360 | 0.627 | 0.194 | 0.008 |
|  | *Thermodesulfobacteria;Thermodesulfobacteriales;Thermodesulfobacteriaceae;Thermosulfurimonas* | | 0.022 | 0.060 | 1.044 | 0.008 |
| Sum of S oxidation and sulfate reduction | | | 49.653 | 40.742 | 46.277 | 7.636 |
| Ammonia oxidation | *Gammaproteobacteria;Chromatiales;Chromatiaceae;Nitrosococcus* | | 1.196 | 0.115 | 0.375 | 1.868 |
| Nitrite oxidation | *Nitrospira;Nitrospirales;Nitrospiraceae;Nitrospira* | | 0.002 | 0.005 | 0.026 | 0.992 |
| Nitrate reduction | *Epsilonproteobacteria;Nautiliales;Nautiliaceae;Nitratifractor* | | 0.489 | 0.105 | 0.062 | 0.057 |
| Nitrification | *Betaproteobacteria;Nitrosomonadales;Nitrosomonadaceae;Nitrosomonas* | | 0.002 | 0.010 | 0.004 | 1.418 |
| N fixation | *Alphaproteobacteria;Rhizobiales* | | 5.739 | 0.562 | 0.450 | 2.622 |
| Sum of ammonia, nitrite oxidation, nitrification and N fixation | | | 7.428 | 0.797 | 0.917 | 6.957 |
| H oxidation | *Aquificae;Aquificales;Aquificaceae;Hydrogenobacter* | | 0.028 | 0.080 | 1.908 | 0.016 |
|  | *Aquificae;Aquificales;Hydrogenothermaceae;Persephonell* | | 0.072 | 0.261 | 4.958 | 0.033 |
|  | *Epsilonproteobacteria;Campylobacterales;Hydrogenimonaceae;Hydrogenimonas* | | 0.290 | 0.025 | 0.198 | 0.008 |
| Sum of H oxidation | | | 0.39 | 0.366 | 7.064 | 0.057 |
| CH4 oxidation | *Gammaproteobacteria;Methylococcales;Methylococcaceae;Methylothermus* | | 1.122 | 0.161 | 2.318 | 0.057 |
| Fe(II) oxidation | *Zetaproteobacteria;Mariprofundales;Mariprofundaceae;Mariprofundus* | | 1.295 | 2.468 | 1.084 | 0.705 |
| Fe(III) reduction | *Deltaproteobacteria;Desulfuromonadales;Desulfuromonadaceae;Desulfuromusa* | | 0.238 | 1.801 | 0.591 | 0.041 |
| Sum of Fe(II) oxidation and Fe(III) reduction | | | 2.655 | 4.43 | 3.993 | 0.803 |
| Mn oxidation | *Alphaproteobacteria;Rhodobacterales;Rhodobacteraceae;Roseobacter* | | 0.068 | 1.234 | 0.353 | 0.049 |
| Total sum in bacteria | | | 60.194 | 47.569 | 58.604 | 15.502 |
| **Archaea** | | | | | | |
| Sulfate reduction | *Crenarchaeota;Thermoprotei;Desulfurococcales* | 0.538 | | 0.003 | 7.460 | 0.014 |
|  | *Crenarchaeota;Thermoprotei;Thermoproteales* | 0.326 | | 0.003 | 1.111 | 0.004 |
| Ammonia oxidation | *Thaumarchaeota* | 45.667 | | 1.062 | 24.636 | 42.745 |
| Total sum in archaea | | 46.531 | | 1.068 | 33.207 | 42.763 |

*The relative abundance in sequencing library is for each sample’s Miseq data set. Taxa are designated by class (phylum for *Crenarchaeota and Thaumarchaeota*), order, family, and genus.

**Figure legends：**

Figure 1 Schematic location of the sampling sites and Longqi vent Field (red Pentagram) at SWIR. The map was created using python

Figure 2. Taxonomic relative abundance of archaeal classes observed of chimney samples for Longqi vent field at SWIR. Bar charts show the Kingdom;Phylum distribution for taxonomically assigned tags that occurred more than 1000 times.

Figure 3: Taxonomic relative abundance of Thaumarchaeota genera observed in chimney samples. Abbreviations: DHVEG-6, Deep Sea Hydrothermal Vent Group 6. Groups in which no tags were sequenced are indicated with a red asterisk. Groups which were detected below1% are indicated with a blue oplus, between 1% to 2% are indicated with a diamond.

Figure 4. Taxonomic breakdown of bacterial 16S SSU rRNA V4-region tags from each chimney sample. Pie Charts show Phylum;Class;Order distribution (The average relative abundance among samples is over 1%) for taxomomically assigned tags that occurred more than 1000 times; the remaining tag sequences are grouped into “Other”.

Figure 5. Relative abundance of Gammaproteobacteria taxa observed in each chimney sample for Longqi field at SWIR. ‘others’ represents the less abundant genera of Acinetobacter, Sedimenticola, Marinicella, Colwellia, Arenicellaceae, Marine methylotrophic Group 2, Coxiella, Granulosicoccus and unclassified Gammaproteobacteria. Groups which were detected below1% are indicated with a blue oplus, between 1% to 2% are indicated with a blue diamond.

Figure 6. Relative abundance of Epsilonproteobacteria genera observed in each chimney samplefor Longqi field at SWIR. Groups which were detected below1% are indicated with a blue oplus, between 1% to 2% are indicated with a diamond.

Figure 7. Clustering analysis tree of the archaeal community structure of chimneys from SWIR, EPR and MAR. LS7=Lucky Strike. Lucky Strike vent field located at MAR (Flores et al., 2011); CH7=chimney sample from EPR, this study.

Figure 8. Clustering analysis tree of the bacterial community structure of chimneys from SWIR, EPR and MAR. LS7=Lucky Strike. Lucky Strike vent field located at MAR (Flores et al., 2011); CH7=chimney sample from EPR, this study

Figure S1. Photographs of hydrothermal vent samples collected from the Longqi field at SWIR.

Figure S2. X-ray diffraction patterns of hydrothermal vent chimney samples colleted from the SWIR Longqi vent field. A: JL90; B:JL94D; C:JL94H; D: JL95

Figure S3. Rarefaction and shannon curves of bacterial (A, B) and archaeal (C, D) 16S rRNA amplicon libraries for chimney samples.

Figure S4. Clustering analysis of bacterial communitis with inferred ecologic roles. SOB represents sulfur oxidizing bacterim; SRB, sulfate reducing bacterium; AOB, ammonia oxidizing bacterium; NOB, nitrite oxidizing bacterium, N-fixer, nitrogen fixation bacterium; HOB, hydrogen oxidizing bacterium; MOB, methane oxidizing bacterium; FOB, iron oxidizing bacterium; FRB, iron reducing bacterium; MnOB, manganese oxidizing bacterium; Table S1 provides more details of taxa related.

Figure S5 RDA analysis for the effect of environmental factors on bacterial communities with inferred function. Fe: the component of iron; Temp: the temperature of fluid; Mn:the component of manganese

Figure S6. Venn diagrams showing the estimated OTU (97 identity threshold) richness shared among bacterial (A) and archaeal (B) communities from hydrothermal vent chimneys at Longqi field on SWIR. Shared OTU richness estimated were calculated using the program Qiime (version 1.9.0). Venn diagrams were plotted using the Venn Diagram package of R. Numbers in the Venn diagrams indicate number of OTUs. The table below the Venn diagrams showed the percent of sharing OTUs for each pair within all the four chimney samples.

Table S1 List for bacterial communities involved in the analysis of clustering and RDA

|  | Taxa |  | Taxa |  | Taxa |
| --- | --- | --- | --- | --- | --- |
| SOB1 | *Hydrogenivirga* | SRB5 | *Sulfurospirillum* | MOB | *Methylothermus* |
| SOB2 | *Helicobacteraceae* | SRB6 | *Thermosulfurimonas* | FOB | *Mariprofundus* |
| SOB3 | *Thiohalophilus* | AOB | *Nitrosococcus* | FRB | *Desulfuromusa* |
| SOB4 | *Thiotrichaceae* | NOB1 | *Nitrospira* | MnOB | *Roseobacter* |
| SOB5 | *Piscirickettsiaceae* | NOB2 | *Nitratifractor* |  |  |
| SOB6 | *Ectothiorhodospiraceae* | NOB3 | *Nitrosomonas* |  |  |
| SRB1 | *Thermodesulfovibrio* | N-fixer | *Rhizobiales* |  |  |
| SRB2 | *Desulfatiglans* | HOB1 | *Hydrogenobacter* |  |  |
| SRB3 | *Desulfobacteraceae* | HOB2 | *Persephonell* |  |  |
| SRB4 | *Desulfobulbaceae* | HOB3 | *Hydrogenimonas* |  |  |
