# Supplementary material for: Microbial Community Structure of Deep-sea Hydrothermal Vents on the Ultraslow Spreading Southwest Indian Ridge
Source: Front Microbiol. 2017 Jun 13;8:1012. doi: 10.3389/fmicb.2017.01012 (PMC5468387; doi:10.3389/fmicb.2017.01012)
Supplement: Supplementary file 9 [file DataSheet2.PDF]

Supplementary Material

Microbial community structures of deep-sea hydrothermal vents on the ultraslow spreading Southwest Indian Ridge

Jian DING[1], Yu ZHANG[2], Han WANG[1], Huahua JIAN[1],Hao LENG[1], Xiang XIAO[12*]

*** Correspondence:**

Xiang XIAO
xoxiang@sjtu.edu.cn

1 Figures

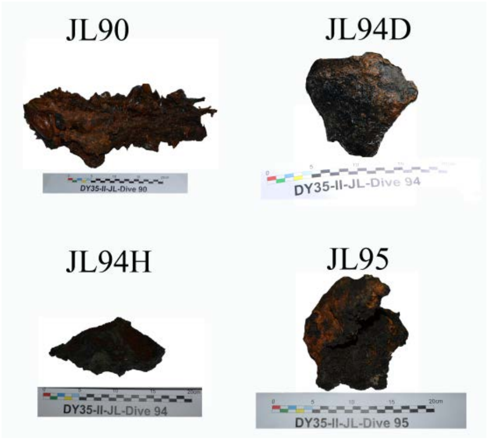

Figure S1. Photographs of hydrothermal vent samples collected from the Longqi field at SWIR.

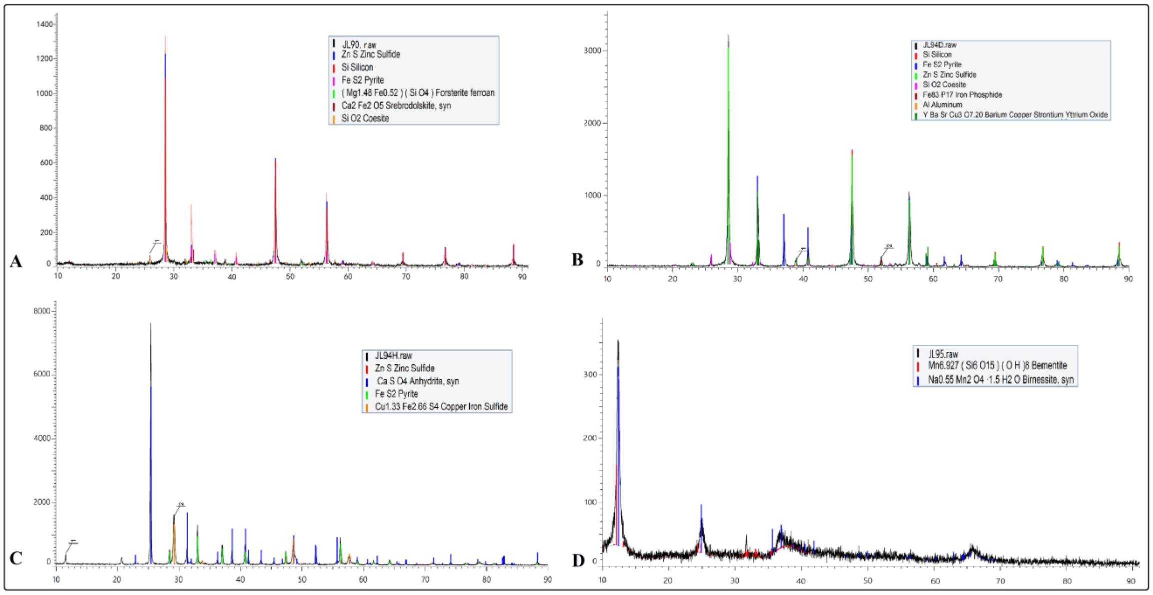

Figure S2. X-ray diffraction patterns of hydrothermal vent chimney samples colleted from the SWIR Longqi vent field.

A: JL90; B:JL94D; C:JL94H; D: JL95

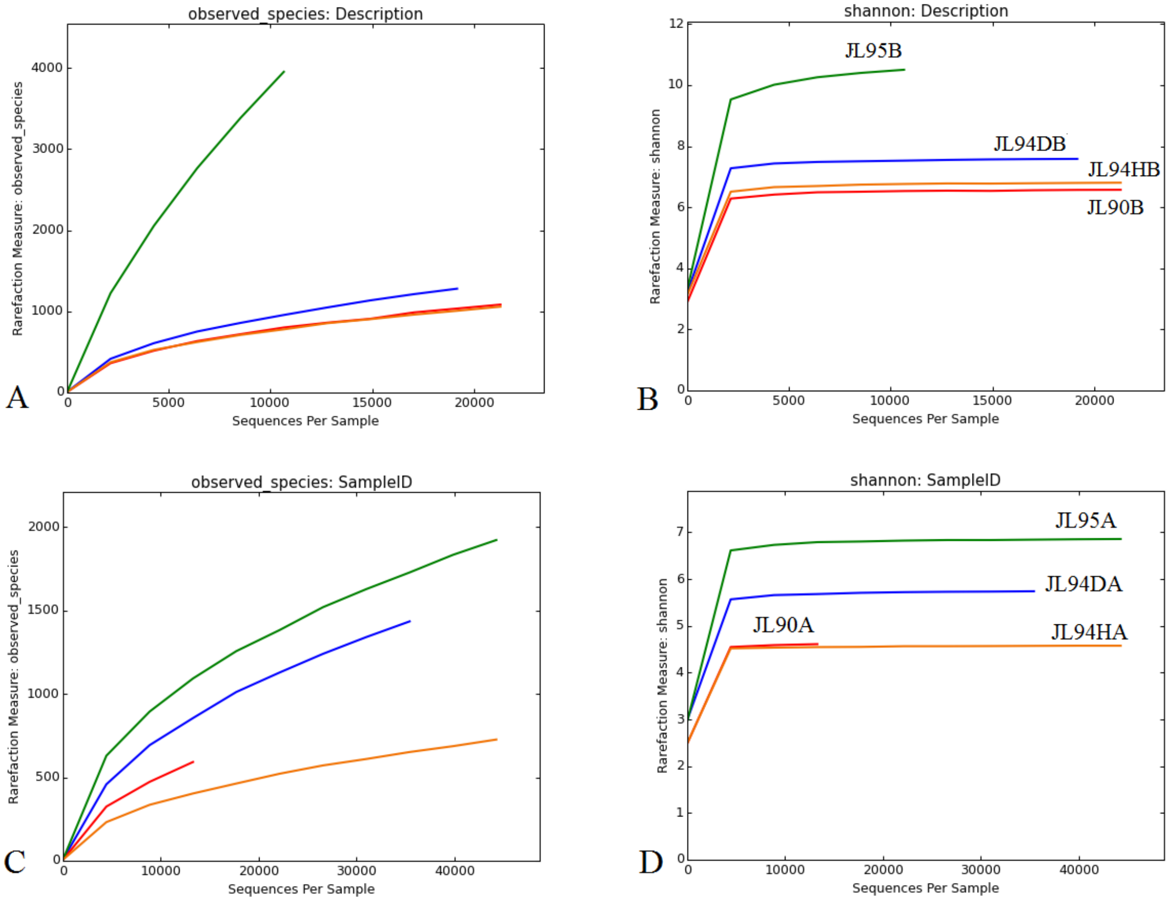

Figure S3. Rarefaction and shannon curves of bacterial (A, B) and archaeal (C, D) 16S rRNA amplicon libraries for chimney samples.

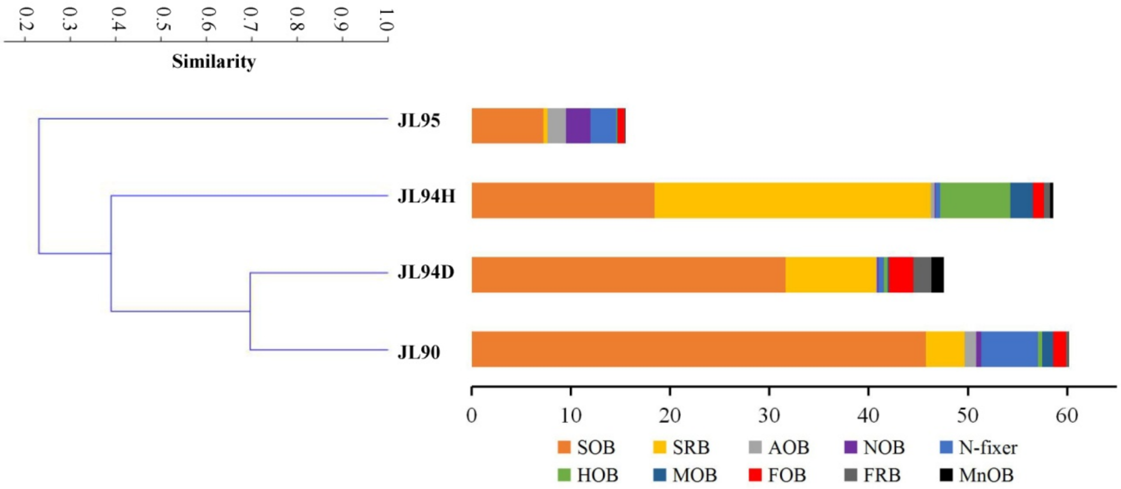

Figure S4. Clustering analysis of bacterial communitis with inferred ecologic roles. Sequence reads that matched a Silva reference database at 97% identity. SOB represents sulfur oxidizing bacterim; SRB, sulfate reducing bacterium; AOB, ammonia oxidizing bacterium; NOB, nitrite oxidizing bacterium, N-fixer, nitrogen fixation bacterium; HOB, hydrogen oxidizing bacterium; MOB, methane oxidizing bacterium; FOB, iron oxidizing bacterium; FRB, iron reducing bacterium; MnOB, manganese oxidizing bacterium; Table S1 provides more details of taxa related.

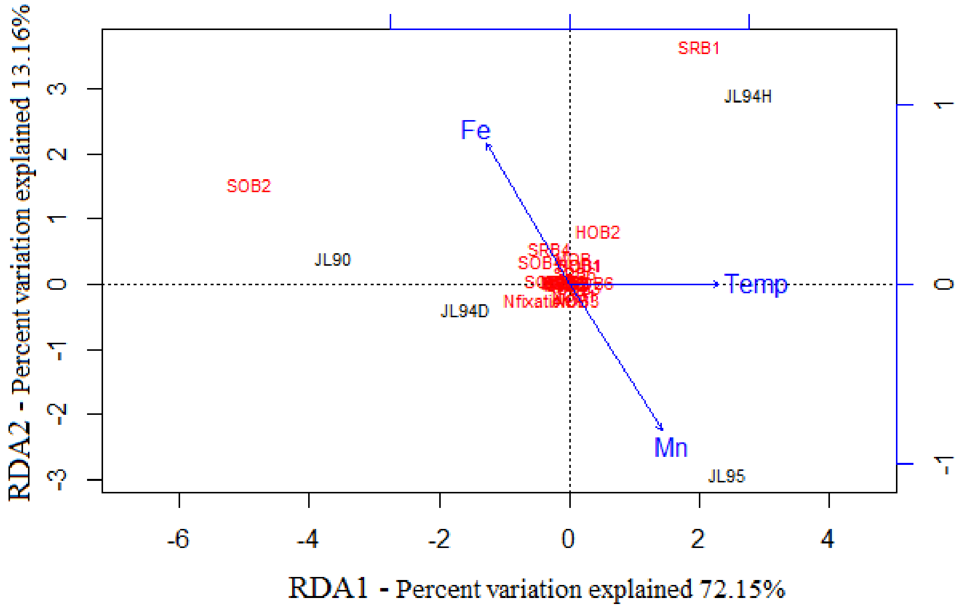

Figure S5 RDA analysis for the effect of environmental factors on bacterial communities with inferred function.

Fe: the component of iron; Temp: the temperature of fluid; Mn:the component of manganese

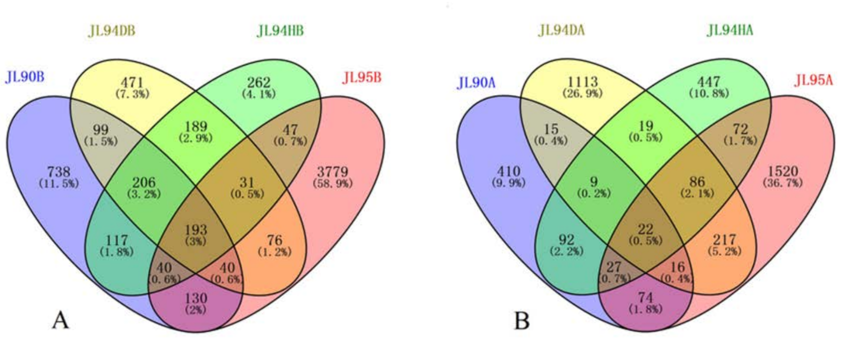

	JL90		JL94D		JL94H		JL95	
	Bacteria	Archaea	Bacteria	Archaea	Bacteria	Archaea	Bacteria	Archaea
JL90	-	-	7.7%	1.1%	7.6%	3.6%	6.2%	3.4%
JL94D	7.7%	1.1%	-	-	9.6%	3.3%	5.3%	14.2%
JL94H	7.6%	3.6%	9.6%	3.3%	-	-	4.8%	5%
JL95	6.2%	3.4%	5.3%	14.2%	4.8%	5%	-	-

Figure S6. Venn diagrams showing the estimated OTU (97 identity threshold) richness shared among bacterial (A) and archaeal (B) communities from hydrothermal vent chimneys at Longqi field on SWIR. Shared OTU richness estimated were calculated using the program Qiime (version 1.9.0). Venn diagrams were plotted using the Venn Diagram package of R. Numbers in the Venn diagrams indicate number of OTUs. The table below the Venn diagrams showed the percent of sharing OTUs for each pair within all the four chimney samples.

2 Table

Table S1 List for bacterial communities involved in the analysis of clustering and RDA

	Taxa		Taxa		Taxa
SOB1	*Hydrogenivirga*	SRB5	*Sulfurospirillum*	MOB	*Methylothermus*
SOB2	*Helicobacteraceae*	SRB6	*Thermosulfurimonas*	FOB	*Mariprofundus*
SOB3	*Thiohalophilus*	AOB	*Nitrosococcus*	FRB	*Desulfuromusa*
SOB4	*Thiotrichaceae*	NOB1	*Nitrospira*	MnOB	*Roseobacter*
SOB5	*Piscirickettsiaceae*	NOB2	*Nitratifractor*		
SOB6	*Ectothiorhodospiraceae*	NOB3	*Nitrosomonas*		
SRB1	*Thermodesulfovibrio*	N-fixer	*Rhizobiales*		
SRB2	*Desulfatiglans*	HOB1	*Hydrogenobacter*		
SRB3	*Desulfobacteraceae*	HOB2	*Persephonell*		
SRB4	*Desulfobulbaceae*	HOB3	*Hydrogenimonas*		
